# Systematic characterization of extracellular vesicle sorting domains and quantification at the single molecule – single vesicle level by fluorescence correlation spectroscopy and single particle imaging

**DOI:** 10.1080/20013078.2019.1663043

**Published:** 2019-09-18

**Authors:** Giulia Corso, Wolf Heusermann, Dominic Trojer, André Görgens, Emmanuelle Steib, Johannes Voshol, Alexandra Graff, Christel Genoud, Yi Lee, Justin Hean, Joel Z. Nordin, Oscar P.B. Wiklander, Samir El Andaloussi, Nicole Meisner-Kober

**Affiliations:** a Department of Laboratory Medicine, Karolinska Institutet, Stockholm, Sweden; b Novartis Institutes for Biomedical Research, Basel, Switzerland; c Imaging Core Facility, Biozentrum, University of Basel, Basel, Switzerland; d Institute for Transfusion Medicine, University Hospital Essen, Essen, Germany; e Department of Cell Biology, Sciences III, University of Geneva, Geneva Switzerland; f Facility for advanced imaging and microscopy, Friedrich Miescher Institute for Biomedical Research, Basel, Switzerland; g Department of Cancer and Stratified Oncology 5, A*star Genome Institute of Singapore, Singapore; h Department of Biosciences, University of Salzburg, Salzburg, Austria

**Keywords:** Extracellular vesicles, exosomes, EV protein sorting domains, EV engineering, fluorescence correlation spectroscopy, single vesicle imaging, CD63, EV double labelling

## Abstract

Extracellular vesicles (EV) convey biological information by transmitting macromolecules between cells and tissues and are of great promise as pharmaceutical nanocarriers, and as therapeutic per se. Strategies for customizing the EV surface and cargo are being developed to enable their tracking, visualization, loading with pharmaceutical agents and decoration of the surface with tissue targeting ligands. While much progress has been made in the engineering of EVs, an exhaustive comparative analysis of the most commonly exploited EV-associated proteins, as well as a quantification at the molecular level are lacking. Here, we selected 12 EV-related proteins based on MS-proteomics data for comparative quantification of their EV engineering potential. All proteins were expressed with fluorescent protein (FP) tags in EV-producing cells; both parent cells as well as the recovered vesicles were characterized biochemically and biophysically. Using Fluorescence Correlation Spectroscopy (FCS) we quantified the number of FP-tagged molecules per vesicle. We observed different loading efficiencies and specificities for the different proteins into EVs. For the candidates showing the highest loading efficiency in terms of engineering, the molecular levels in the vesicles did not exceed ca 40–60 fluorescent proteins per vesicle upon transient overexpression in the cells. Some of the GFP-tagged EV reporters showed quenched fluorescence and were either non-vesicular, despite co-purification with EVs, or comprised a significant fraction of truncated GFP. The co-expression of each target protein with CD63 was further quantified by widefield and confocal imaging of single vesicles after double transfection of parent cells. In summary, we provide a quantitative comparison for the most commonly used sorting proteins for bioengineering of EVs and introduce a set of biophysical techniques for straightforward quantitative and qualitative characterization of fluorescent EVs to link single vesicle analysis with single molecule quantification.

## Introduction

Cell to cell communication by extracellular vesicles (EVs) is gaining attention in basic cell biology research [1–6], biomarker discovery [7–10] and therapeutic drug delivery [11–14]. Thus, there is high demand for an increased mechanistic and quantitative understanding of how EVs interact with recipient cells and similarly, how to best harness EVs as delivery vehicles.

To monitor EV cell uptake and biodistribution, fluorescence-based detection is one of the methods of choice. An important technical challenge is a selective labelling of desired vesicles on the background of other extracellular vesicles and particles, with minimal perturbation of their physicochemical and biological properties. A relatively straightforward and widespread approach is to stain EVs post-isolation with fluorescent lipophilic dyes [14–27] such as DiR [15,20], DiO [21], DiD [22], FM4-64 [23], CFSE [23,24], PKH-26 [25,26] and PKH-67 [14,27,28]. These compounds are lipid-like molecules with fluorescent head groups and long aliphatic tails capable of inserting into the vesicle membrane and have become of extensive use for fluorescence-based studies of EVs [16–19]. The major drawback of lipophilic tracers, however, is that they are not EV specific and indiscriminately stain all lipid-containing particles and vesicles within the sample [29,30]. Likewise, being lipophilic, they can also redistribute from the EVs to the cellular membranes. This complicates the interpretation of biodistribution data due to lack of discrimination of dye-labelled EVs from secondary homing of the dye itself [31,32]. Additionally, in salt-containing buffers or media, most of these lipid stains readily form micelles or aggregates with partially overlapping physicochemical properties to EVs, thereby providing difficulties to be removed and thus a risk for additional artefacts [29]. To overcome these limitations, the field is turning towards more specific and less invasive strategies such as expression of reporter protein-tagged EV surface markers [17,31,33–36] or metabolic biorthogonal conjugation [37] within EV-producing cells. These approaches have been successfully used for visualizing EVs *in vivo* and *in vitro* to shed light on the vesicular biogenesis and biodistribution processes. Similar approaches have been used for engineering EVs with payloads; endogenously expressed proteins, such as Lamp2b, Lactadherin (MFGE8) and in particular CD63, have been exploited as sorting domains to load EVs with RNA [35], cytokines [38], tumour associated antigens [39–41], tissue-specific targeting peptides [11] or as proteins to anchor specific tissue targeting peptides [42]. Yet, it remains unclear whether a non-physiological overexpression of transgenic protein-tagged EV markers within the parent cells may influence EVs biogenesis, release, as well as their composition due to cell stress or simply altered surface marker abundance. The choice of proteins used to modulate EV properties might depend on the final intent, but a comparison of different EV protein markers and their efficiency in sorting moieties into EVs is still lacking. In particular for therapeutic applications, a quantitative basis linking molecule numbers to vesicular concentrations will be of utmost importance for moving into pharmacological studies.

In this report, we provide an extensive characterization of EVs labelled via overexpression of GFP-tagged proteins in parent cells for an array of transmembrane as well as luminal EV marker proteins at the single molecule-single vesicle level. Thereby we provide a reference for choosing EV sorting domains as well as a set of straightforward methodology for quantitative characterization to support EV engineering.

## Results

### CD63-GFP labelling results in minimal vesicle perturbation

**Figure 1.**
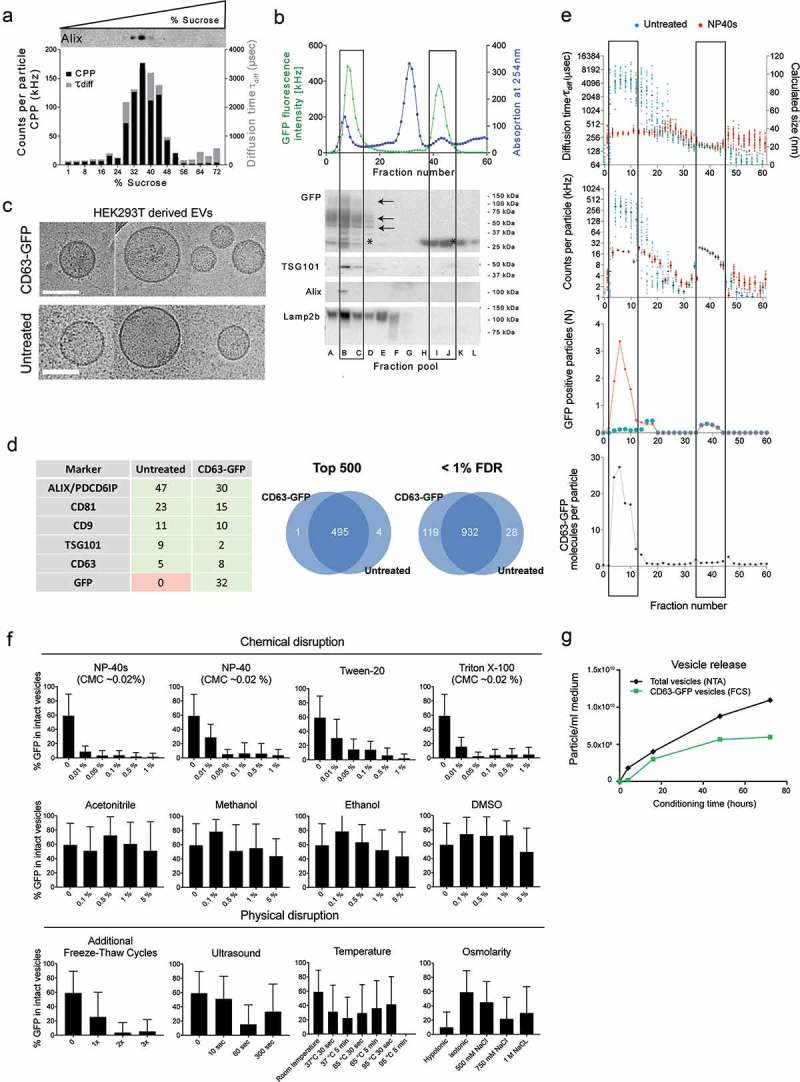
Single molecule – single vesicle characterization of CD63-GFP-tagged extracellular vesicles. (a) Direct sucrose gradient fractionation of conditioned medium from CD63-GFP transfected HEK293T cells without prior ultracentrifugation. X-axis: measured sucrose concentrations in collected fractions. GFP fluorescent particles of ca 50–120 nm diameter (measured by FCS) co-fractionate with the EV marker Alix, peaking at ca 32–36% sucrose. (b) Following UF on a 100 kDa MWCO membrane, enriched medium was loaded onto a Superdex200 column for size exclusion chromatography, with continuous UV detection (blue line). The total GFP fluorescence count rate was measured in each of the 60 fractions by FCS (upper panel, green line). Western blots for different exosomal markers were performed after pooling of 4 fractions each into samples A-L as indicated, omitting one fraction in between pools. CD63-GFP fusion protein bands (multiple glycosylated isoforms) versus truncated GFP bands detected on an anti-GFP western blot are indicated by arrows and asterisks, respectively. (c) Cryo-EM analysis of UF-SEC isolated CD63-GFP versus native EVs (representative images). Scaling bar: 100 nm. (d) Left panel, LC-MS proteomics of native versus CD63-GFP HEK293T EVs. Spectral counts for a subset of proteins from the list in Table S1 are shown. Right panel, Venn diagram comparing the top 500 proteins, ranked by total spectral count (left), or all proteins detected at a < 1% false discovery rate (FDR) (right). (e) Individual fractions from size exclusion chromatography of HEK293T CD63-GFP EVs in Figure 1(b) were analysed by FCS with (red data points) and without (blue data points) vesicle disruption by the detergent NP40s. The translational diffusion time (τ_diff_, upper panel left y-axis and conversion into hydrodynamic diameter, right y-axis), molecular brightness (CPP, second panel) as well as absolute number of fluorescent molecules within the confocal volume (N, third panel) are depicted across the fractions. The ratio of freely diffusing GFP fluorescent particles before and after NP40s treatment (third panel) yields the average number of CD63-GFP molecules per particle (bottom panel). Frames indicate fractions comprising the two main fluorescent populations: the first indicates a heterogenous population comprising mostly GFP-tagged EVs, whereas the second one consists of monomeric GFP. (f) Disruption of EVs (stored at −80°C) with different mechanical and biochemical treatments based on the fraction of intact vesicles versus free GFP measured by FCS. (g) Time course of CD63-GFP vesicle release. The concentration of CD63-GFP positive vesicles in UF enriched samples from HEK293T cells was determined by FCS, the total number of vesicles between 70 and 140 nm in size was determined by NTA. Time points represent hours of conditioning, starting 5 h after transfection.

CD63 was the first protein to be characterized as part of the tetraspanin family and found to be expressed at the cell surface, in the endosomal compartments and in exosomes [43]. It is also known to have a number of interaction partners such as integrins [44], syntenin [45] and other members of the tetraspanin [46] family which might be involved in the EV biogenesis and vesicular protein sorting. To retain natural functions of CD63 in signal transduction or integrin complexation, GFP was fused to the N-terminus of CD63, thereby oriented to the cytosolic side of the EV membrane. CD63-GFP was transiently overexpressed in HEK293T cells and EVs were isolated 48 h later. First, EVs were separated by sucrose fractionation. Exosomes have been well established to float at 33–39% (w/v) sucrose (corresponding to 1.13–1.19 g/ml) [47], although the gradient fractionation has so far been done following at least one initial ultracentrifugation [47] or ultrafiltration step [48], to the best of our knowledge. To analyse the vesicles in their native state without any prior concentration steps that might affect their physiochemical properties, we fractionated conditioned medium (CM) from transfected HEK293T cells directly onto a sucrose cushion with a steep gradient. This confirmed that the EV markers Alix and Tsg101 sedimented at a density of 32–40% (1.13–1.18g/mL) sucrose without any prior concentration step (Figure 1(a)) and at a similar sucrose density as EVs from untransfected cells (Figure S1(a)). Fluorescence correlation spectroscopy (FCS) analysis in all fractions further confirmed the enrichment of GFP positive particles with translational diffusion times (τ_diff_) of 2–10ms, corresponding to a vesicle size of ca 50–120 nm and co-fractionating with Alix (Figure 1(a)). CD63 itself was not detected by western blotting in the gradients from unconcentrated medium, most likely due to limited antibody sensitivity.

To next isolate CD63-GFP vesicles in as native state as possible, a successive ultrafiltration-size exclusion chromatography (UF-SEC) protocol was used [49]. Briefly, conditioned medium was pre-spun at low centrifugal forces, filtered (0.22 μm pore size), concentrated by ultrafiltration on a 100 kDa MWCO spin-filter and fractionated by size exclusion chromatography on a Superdex-200 size exclusion chromatography column (Figure 1(b)). Consistent with our previous description of EV isolation by SEC [49], the UV chromatogram revealed two major peaks, the first corresponding to the expected elution volume of vesicles (ca 1 MDa, Figure S1(c)), the second at circa 82 kDa most likely comprising residual serum albumin and other serum proteins, as supported by Bradford protein quantification (Figure S1(b), upper panel). In line with the first peak comprising vesicles and the second peak comprising non-vesicular protein complexes, the ratio of 254/280 absorption was also markedly different with Abs_254_:Abs_280_ ~ 1.3 in peak 1, and Abs_254_:Abs_280_ ~ 2 in peak 2 (Figure S1(b), bottom panel). Pooled samples of 4 fractions each (omitting one fraction between pools) were analysed by western blotting (Figure 1(b)). The exosome marker proteins Alix and Tsg101 eluted in a relatively sharp peak close to the void volume, as expected based on the upper size separation limit of the column at 600 kDa (exclusion limit 1.3 MDa). The widely used EV marker Lamp2b also co-eluted with the vesicles, however, showed a broad elution profile with two apparent maxima, suggesting that it might in part be associated with a different vesicle population. The CD63-GFP fusion protein peaked with the exosome markers, however, a second major GFP fluorescent peak was observed eluting in later fractions and comprising GFP truncated from CD63 according to western blotting (Figure 1(b)). The fractions of peak 1 were pooled, concentrated and used for further characterization (“UF-SEC” EVs). In addition to the size and density, we next assessed the morphological integrity of EVs from CD63-GFP transfected parental cells. UF-SEC enriched EV samples with and without fluorescent markers were analysed by cryo-electron microscopy (cryo-EM). In both conditions, the samples contained a similar repertoire of predominantly double membrane enclosed, round, protein-coated vesicles with relatively homogeneous structure and size of ca 50–140 nm in diameter (Figure 1(c)). To determine whether CD63-GFP parent cell transfection might result in significant changes of the EV protein content, UF-SEC enriched EVs from CD63-GFP transfected versus non-transfected HEK293T cells were analysed by MS-proteomics (Table S1), confirming a high abundance of several canonical exosomal markers (Figure 1(d)). MS-proteomics analysis (Figure 1(d) and Table S1) showed that the protein composition of HEK293T CD63-GFP vesicles closely matched that of the native (untreated) cells. In fact, among the 500 most abundant proteins (based on spectra count), GFP was essentially the only different cargo found in vesicles derived from the CD63-GFP transfected cells. Even when including all confidently identified proteins (cut-off at 1% false discovery rate), the overlap was still around 90%, which is in the range of what is generally expected for replicate-unbiased MS-proteomics analyses in samples of similar complexity [50]. Interestingly, the proteomic data also showed similar spectral count values for CD63 in EVs derived from CD63-GFP transiently transfected cells compared to untransfected cells. This suggests comparable abundance levels in the EVs, despite non-physiological overexpression in the cells.

### Single molecule – single vesicle characterization by fluorescence correlation spectroscopy

To characterize the GFP labelled vesicles at the single molecule – single vesicle level, we set up a workflow for analysis by Fluorescence Correlation Spectroscopy (FCS). FCS has been introduced by Rudolf Rigler, Watt Web, and co-workers in the 1970ies [51,52] and is based on single photon sensitive detection of temporal fluorescence fluctuations in a defined confocal volume. The autocorrelation of the fluctuating signal is used to derive molecular parameters such as translational diffusion times (related to sizes), molecular brightness and absolute particle numbers. Due to the single molecule sensitivity and diffusion-based detection, it is applicable to track particles below the Abbe diffraction limit. In contrast to many other currently used technologies for EV tracking, it does not have any size bias. We first analysed all fractions from the SEC by FCS to quantitatively characterize the GFP positive species within these two major fluorescent peaks (Figure 1(e)). Fractions of peak 1 comprised predominantly a population of GFP fluorescent, large particles with relatively heterogeneous translational diffusion times ranging from ca 2–10ms (mean: ~4 ms), corresponding to ca 50–120 nm in diameter (mean: ~100 nm). A similar heterogeneity was observed in molecular brightness (counts per particle, CPP), ranging from ca 80 kHz to 1000 kHz per particle (mean of ca 250 kHz per particle). To further resolve and quantify the association of CD63-GFP with EVs we next intended to identify conditions for mild disruption of the vesicles compatible with spectroscopy and allowing for solubilisation of membrane proteins (Figure 1(f)). Among a set of different chemical and physical treatments tested, the non-ionic surfactant NP40s displayed the highest level of vesicular disruption at a concentration below the critical micelle concentration (Figure 1(f)). Treatment with NP40s resulted in a uniform population of molecules with a translational diffusion time of 300 μs, corresponding to the expected τ_diff_ of free CD63-GFP (based on extrapolation from data measured for free GFP, τ_diff_ = 160 μs, data not shown). These data confirm a physical association of CD63-GFP with detergent sensitive particles. Consistent with a presence of multiple CD63-GFP molecules per vesicle, NP40s treatment resulted in a drop of the molecular brightness of the particles, yielding a homogeneous population of molecules with similar brightness as measured for free, monomeric GFP (18 kHz). The number of GFP molecules independently diffusing through the confocal volume was in turn increased upon vesicle disruption by NP40s. Together, these data allow to quantify the numbers of CD63-GFP per vesicle, which ranged between 10 and 30 molecules per individual vesicle (Figure 1(e)). In contrast, the second major eluting GFP peak (fraction 40–45) comprised molecules of relatively homogeneous translation diffusion times and molecular brightness, insensitive to NP40s treatment and consistent with the size and brightness expected for monomeric GFP. These data are in line with the western blotting results in Figure 1(b) and corroborate that this second species contains mainly non-vesicular, truncated GFP. Similar data were obtained for Huh7- and B16F10-derived EVs (Figure S1(d)). Based on this characterization, the fractions comprising EV markers and GFP positive vesicles were pooled after SEC and are henceforth referred to as UF-SEC EVs.

Due to this background of free, non-vesicular GFP found in conditioned medium, we would like to point out that bulk GFP fluorescence is not a reliable measure of EV release. However, FCS allowed to specifically quantify vesicular GFP within the heterogeneous samples. We therefore reasoned that it might be a straightforward method to directly quantify the release of CD63 positive vesicles within conditioned medium, despite the background of free, non-vesicular GFP. For sensitivity reasons, we concentrated the conditioned medium by ultrafiltration (100 kDa MWCO) and then determined the concentration of CD63-GFP vesicles released at different time points by FCS. We used a 2-component fit with a diffusion time of free, non-vesicular GFP set to the measured value of 160 µs, which was further consistent with the GFP diffusion time measured in the flow-through from the UF (data not shown). This allowed to specifically determine the concentration of GFP positive vesicles as well as free GFP. The total number of vesicles per ml of conditioned medium as determined by Nanoparticle Tracking Analysis (NTA) increased over time, whereas the release of fluorescent vesicles quantified by FCS was delayed by several hours, most likely reflecting the onset of expression in transfected cells (Figure 1(g)). The relative fraction of CD63-GFP vesicles was highest after 16 h and reached up to an apparent 80% of all vesicles. While both, fluorescent and total vesicle numbers further increased over time, the relative fraction of fluorescent vesicles declined at prolonged conditioning time, which might be due to the transient reporter plasmid being diluted out with cell division, and/or an increased contribution of other types of vesicles being released at later time points.

### Characterization of different GFP-tagged EV proteins in HEK293T cells

**Figure 2.**
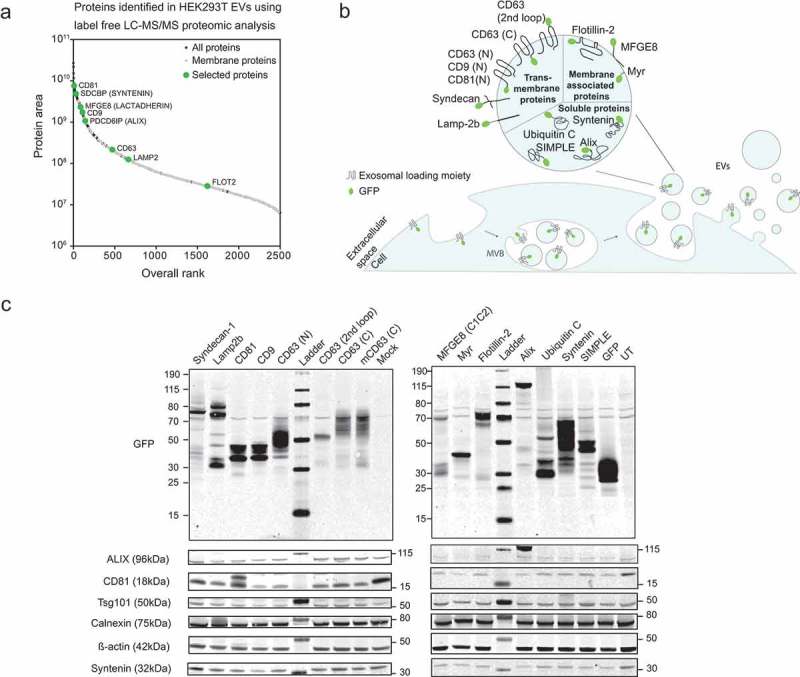
Overexpression of different GFP-tagged EV-markers in HEK293T parental cells. (a) LC-MS/MS proteomic analysis of HEK293T-EVs. The overall protein rank (X-axis) is plotted against the protein area expressed in base-10 log scale (Y-axis). Proteins which have been selected for the screening are marked in green. (b) Schematic representation of EV biogenesis with a zoomed-in vesicle depicting the proteins selected as loading moieties and their theoretical vesicular localization. (c) Western blot on HEK293T cells illustrating the molecular weight (kDa) of each GFP-fused protein (upper panels) and the expression of several EV markers across the samples (lower panels). β-actin was used as loading control.

Following the characterization of CD63-GFP fluorescent EVs, we proceeded to test a selection of other proteins as potential EV tagging or sorting proteins. For this purpose, 12 proteins known to be present in EVs were engineered as GFP-fusion constructs (Figure 2(b) and S2). This included CD9 [53,54], CD81 [53,54], Syntenin [53,55] and Alix [55] which have been widely used as EV markers [56] and were also detected among the 500 most abundant proteins in the LC-MS/MS analysis on HEK293T EVs (Figure 2(A) and Table S2). From this list, we additionally selected the C1C2 domain of MFGE8 and Lamp2b since they have been previously used as sorting domains to load EVs with tumour-associated antigens [39–41] and tissue-specific targeting peptides [11] respectively. Further candidates were selected based on their previously reported relation to exosome biogenesis: Syndecan was shown to interact with Alix through Syntenin and to support EV biogenesis [55]; Flotillin-2, a commonly used EV marker [57], is implicated in endocytic mechanisms [58]; SIMPLE (Small Integral Membrane Protein of the Lysosome/late Endosome) is a protein causing an inherited demyelinating neuropathy once mutated [59] and was found in intraluminal vesicles (ILVs) of MVBs and exosomes [60]. We further included a Myristoylation tag (Myr) [61] as a plasma membrane anchor and the Ubiquitin C tag [62,63], which have both been reported to target cytoplasmic proteins to the EV membrane. Untagged GFP was used as a reference.

The coding sequences were fused to the GFP open reading frame (ORF) and cloned into an expression vector under the control of the CMV promoter (Figure S2; for cloning strategy see Table S3). The GFP fusions were generated either at the N- or C-terminus to best retain each protein’s natural function based on previously- published studies and from a protein database research. For CD63, different integration sites were assessed: N- and C-terminal fusions should both result in cytoplasmic orientation of GFP; integration into the second loop of the tetraspanin should result in presentation at the EV surface. The different protein encoding vectors were transiently overexpressed in HEK293T cells using Polyethylenimine (PEI) transfection for 4 h followed by serum-free media change. Cells were collected 48-h post transfection, pelleted and analysed by flow cytometry. The cell viability after transfection was comparable to non-transfected cells regardless of the protein expressed (above 90%, Figure S3(e)). The transfection efficiency was evaluated as the percentage of viable/single/GFP positive cells by flow cytometry, which ranged from 20% to 40% across the samples (Figure S3(f)).

To match the fluorescence to protein levels, transfected HEK293T cells were lysed and the protein expression verified by western blotting (Figure 2(c)). All samples were probed for GFP, confirming the predicted molecular weight for each chimeric protein. In most of the samples, a band around 30 kDa was detected, again indicating a possible cleavage of GFP from the rest of the protein. The expression levels of the EV markers Alix, Tsg101 and Syntenin were similar in the source cells from all samples and analogous to untreated cells, only CD81 exhibited variable expression levels (Figure 2(c)). Probing for the EV markers Alix, Syntenin, CD81 and Lamp2b allowed the detection of both the endogenous and the bioengineered proteins in the respective overexpressed samples, with a size shift corresponding to the GFP fusion (Figure S4). Calnexin was used as a non-EV control protein.

### Vesicular loading of GFP is more efficient when coupled to membraneous as opposed to cytosolic proteins

We next aimed to quantify the EV sorting capacity of the 12 selected GFP-tagged target proteins using different biophysical methods. A simplified SEC protocol was used (“UF-qEV”) to obtain sufficient throughput for isolating EVs side by side. HEK293T cells were transfected with the individual GFP-expression constructs. Medium was conditioned for 48 h, pre-cleared as above and concentrated by UF (100kDa MWCO spin filter). The retentate was then loaded on a qEV size exclusion column (Izon) to fractionate the EV and non-EV components. To relate this method to the results obtained using UF-SEC (Figure 1(b)), we first analysed individual fractions (0.5 ml) of the CD63-GFP samples by immunoblotting, NTA and total protein quantification (Figure S5). The EV markers Alix and CD81 eluted in fractions 7 to 14 along with CD63-GFP labelled EVs (Figure S5(a)), followed by the protein peak (fractions 13 to 27) as shown by the DC protein assay (Supplementary Figure 5C, blue line) and Ponceau total protein staining (Supplementary Figure 5B). CD81 was detected in both EV containing fractions as well as co-eluting with the protein peak. Based on these data and in correlation with the particle peak detected by NTA (Supplementary Figure 5C, black line), fractions 7 to 11 were selected as EV enriched fractions and largely devoid of non-EV proteins. These fractions were pooled and further concentrated using a 10 kDa MWCO spin filter. Alike for UF-SEC (Figure 1(b)), a truncated form of GFP was also detected within the EV fractions, but no additional peak was observed for free GFP, likely eluting outside the collected range (Figure S5(a)).

**Figure 3.**
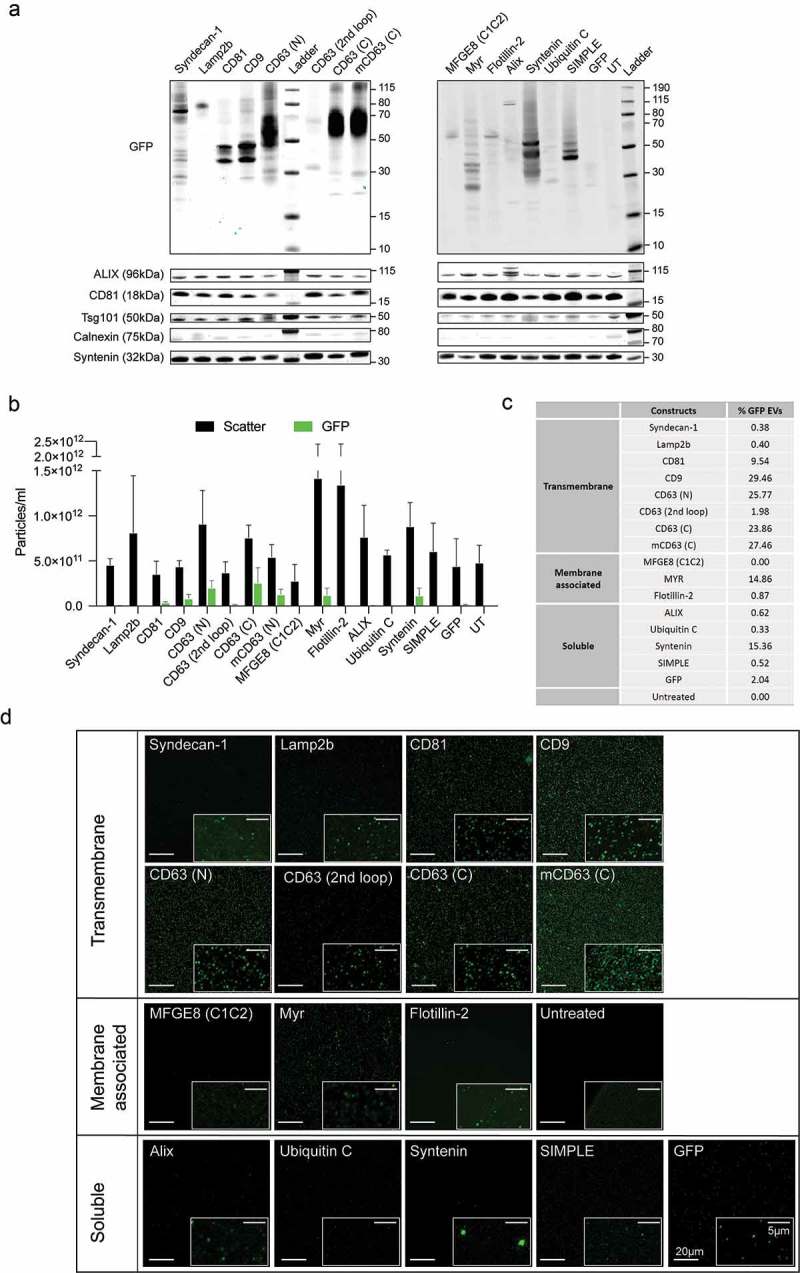
Loading of different GFP-tagged EV markers into UF-qEV purified EVs. (a) Western blot of HEK293T-derived EVs illustrating the molecular weight (kDa) of each GFP-fusion protein (upper panels) and the expression of several EV markers across the samples (lower panels). (b) NTA analysis of isolated EVs showing the total particle concentration (black) and the concentration of GFP positive particles (green) in each sample (N = 3). (c) Table representing the percentage of GFP positive vesicles over total particle count (N = 3). (d) Single vesicle imaging using confocal fluorescence microscopy after spotting EVs onto coverslips (scale bar in the left-bottom corner is 20 µm, all overview images are shown to scale). Individually contrast-enhanced zooms are shown for illustration in the right-bottom corner (scale bar in the upper-right corner is 5 µm).

Using the UF-qEV method, we then isolated EVs derived from HEK293T cells transiently transfected with the different chimeric EV sorting proteins. The protein levels of endogenous Alix, Tsg101 and Syntenin were relatively comparable across the different samples regardless of the overexpressed protein, whereas CD81 again exhibited varying expression levels (Figure 3(a)). Both endogenous and engineered proteins were identified when probing the samples from the corresponding constructs (asterisks in Figure S6(a and b)). Calnexin was detectable at low levels in all samples including the non-transfected control (UT) (Figure 3(a)). Based on GFP-immunoblotting (Figures 3(a) and S6(d)) most of the transmembrane proteins were recovered in EVs, with the exception of CD63 (2^nd^ loop). For membrane-associated and soluble markers, the highest levels of chimeric proteins in EVs were observed for Myr, Syntenin and SIMPLE. A truncated form of GFP was again detected in most of the EV samples. Quantification of the GFP intensity of the relevant bands in both cells and EVs is shown in Figure S6(d). Using NTA, the percentage of GFP fluorescent particles was determined based on the ratio of the concentration of fluorescent vs scatter particles (Figure 3(b and c)). In addition, we used confocal fluorescence microscopy to image fluorescent vesicles after spotting them onto coverslips. For most constructs, we were able to detect individual light diffraction-limited fluorescent spots corresponding to single vesicles [34] (Figure 3(d)). Both, brightness and number of fluorescent particles in the field of view (FOV) were largely consistent with the NTA and western blotting data, with the exception of Syntenin that revealed the presence of a small population of very bright particles (Figure 3(d)). Together, these data indicated efficient vesicular tagging when GFP was coupled to CD81, CD9, CD63 (N) and (C), Myr and Syntenin, ranging up to ~25% of GFP positive particles. Low levels of GFP-fusion proteins as well as GFP fluorescence were observed for EVs from CD63 (2^nd^ loop), Syndecan-1, Lamp2b, MFGE8/C1C2, Alix, UbiquitinC and SIMPLE GFP-chimera. These constructs were comparable to overexpression of untagged GFP which resulted in the recovery of a small fraction of GFP positive vesicles (ca 2%). To complement these data, we next applied Imaging Flow Cytometry (IFCM) which combines flow cytometry with image-based signal quantification, with settings that were recently qualified and optimized for the quantitative analysis of single EVs by using CD63-GFP labelled EVs as biological reference material [33]. Here, the optimized settings were applied to measure the concentration of GFP positive EVs in conditioned media samples derived from cells transfected with the different GFP constructs at the single vesicle level (Figure S7(a–d)). Consistent with what was observed by confocal imaging, the tetraspanins CD9, CD63 (N-, C- and mCD63), CD81, as well as Myr and to a smaller extent Syntenin, showed the highest number of GFP positive EVs per culture volume.

### Quantitative characterization of EVs with different GFP reporters at the single vesicle level

**Figure 4.**
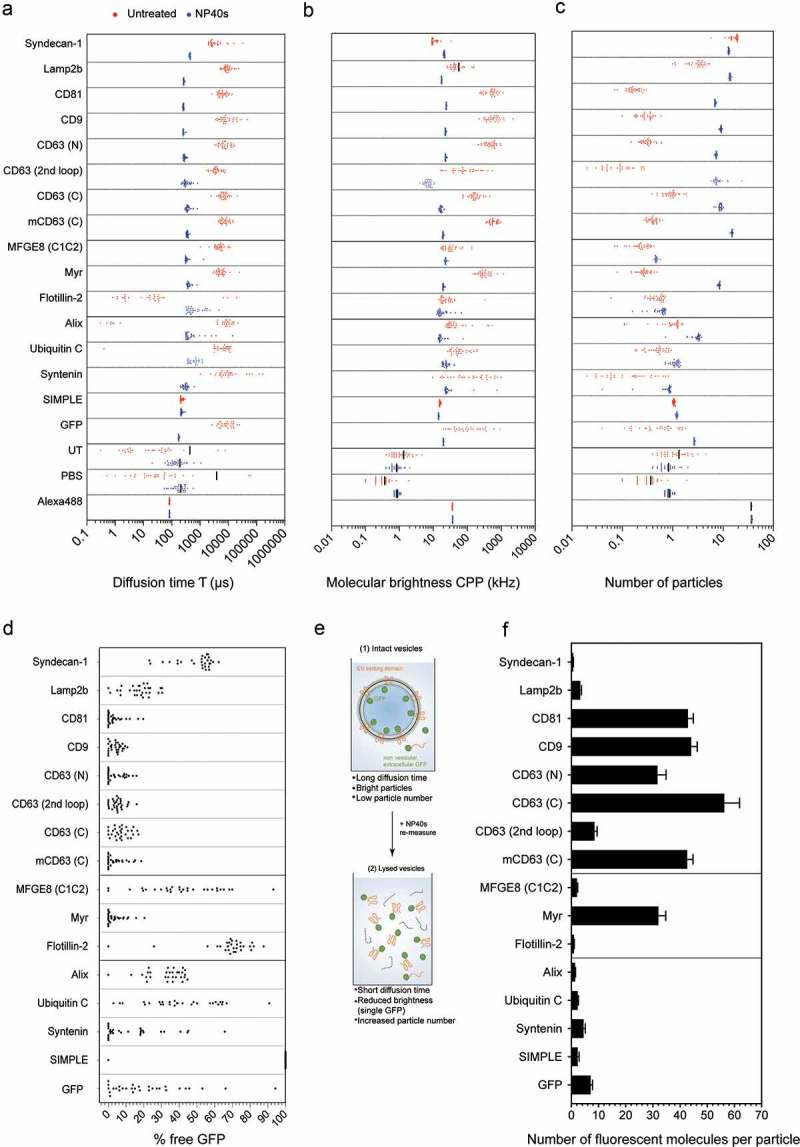
Fluorescence correlation spectroscopy of different GFP-tagged EVs. UF-qEV isolated vesicles, carrying different EV proteins fused to GFP were analysed by FCS without (red data points) and with vesicular disruption by NP40s (blue data points). (a) The diffusion time τ (µs), (b) the molecular brightness CPP (kHz) and (c) the number of particles is shown for each GFP-tagged EV protein. (d) The percentage of freely diffusing GFP fluorescent particles was calculated based on a 2-component FCS analysis using the τ_diff_ of free versus vesicular GFP. (e) Schematic overview of the vesicular disruption by NP40s treatment and the relative observed parameters. (f) Average number of GFP molecules per vesicle based on increase in particle numbers and decrease in molecular brightness upon vesicle disruption by NP40s. Bar graphs indicate representative biological replicates (individual biological replicates are shown in Supplementary Figure 8(b)). Error bars represent standard deviations from at least four technical replicates.

We next characterized the EVs from all constructs with FCS (Figure 4 and Figure S8). As above, disruption of the vesicles with NP40s was used to determine the number of fluorescent molecules per vesicle (Figure 4(e)). First, we measured the translational diffusion time of each of the solubilised GFP-tagged proteins (Figure 4(a)). This value was then used for the free GFP-fusion protein fraction within a 2-component fit of the corresponding intact EV samples to determine the fraction of free versus vesicular GFP-reporter in the non-NP40S sample (Figure 4(d)). The translational diffusion time of the vesicular fraction further provided a measure for the size distribution of the particles carrying GFP. The ratio between the molecular brightness of the intact EVs and free GFP molecules, determined with and without NP40s lysis (Figure 4(b)), allowed to derive the numbers of GFP-tagged proteins per vesicle. The change in total fluorescence intensity was used to calculate a correction factor for GFP fluorescence unquenching upon EV disruption. As an alternative measure for the GFP molecules per vesicle, we used the increase in freely diffusing GFP molecules upon NP40s disruption (Figure 4(c)) which was generally in good agreement with the decrease in molecular brightness. We then derived an average of the values determined by these two complementary methods, and from at least four technical replicates (Figure 4(f) and Supplementary Figure 8(b)). The highest abundance in EVs was observed for the GFP-tagged tetraspanins which ranged up to ca 40–60 molecules per fluorescent vesicle, showing a relatively homogeneous population of GFP fluorescent vesicles with translational diffusion times corresponding to the expected size range of ca 80–120 nm (confer to Figure 1(e) for conversion of τ_diff_ to calculated hydrodynamic diameter). For the different CD63 constructs the abundance showed significant differences with highest numbers observed for N-terminal fusion constructs, indicating that vesicular sorting of CD63 is sensitive to the tagging with GFP, in particular when inserted into one of the extracellular loops. Mouse CD63 was efficiently sorted into EVs also in human cells, indicating functional conservation of its vesicular sorting across species. The single-pass transmembrane receptor Lamp2b was present in vesicles of a similar size as the tetraspanins but at lower levels, which is consistent with the western blotting and confocal imaging data in Figure 3. Syndecan was present in a vesicular population at an average of ~1 molecule per vesicle but these particles were smaller in size according to the translational diffusion time and showed a quenched molecular brightness. From the tested membrane-anchored or -associated proteins, fusion to the Myr tag resulted in efficient sorting of GFP into EVs with an abundance close to the tetraspanins, and a relatively homogeneous population of bright vesicles in a similar size range as CD63 vesicles. In contrast, the phosphatidylserine binding MFGE8/C1C2 reporter resulted in only a small population of GFP positive vesicles which was in the expected size range but highly variable in molecular brightness and numbers of GFP, averaging at 2–3 molecules per vesicle. GFP-tagged Alix resulted in a major population of non-vesicular GFP with a small fraction loaded into vesicles of the expected size and with an average of 3–4 molecules per vesicle. Interestingly, Alix-GFP was not significantly enriched in EVs as compared to untagged GFP. No vesicular loading was observed for GFP when fused to the SIMPLE tag or to Flotillin-2. The data were in good agreement with the single vesicle imaging results in Figure 3 and even though a small fluctuation was observed between different EV batches due to transfection and isolation variability, the data were well reproduced among several independent measurements (shown in Figure S8(b–d)).

### Co-expression of different GFP-tagged EV proteins with CD63-mCherry at the single vesicle level

An increasing number of studies have shed light on the heterogeneity of EVs, providing evidence for the existence of different subpopulations with unique RNA signatures [64], protein profiles [53] and biological functions [65]. These data suggest that the heterogeneity of the EVs might involve distinct or mixed biogenesis processes allowing for sorting of diverse cargos into different EV subpopulations. To investigate the specific sorting of the different proteins into CD63-GFP positive EVs, we transiently co-expressed the GFP-tagged EV proteins with CD63-mCherry in HEK293T cells and isolated the EVs via UF-qEV (Figure 5). The expression of each protein in co-transfected HEK293T cells was validated by western blotting (Figure S9). An equal number of cells was lysed and loaded on the gel, as verified by the uniform β-actin expression. As shown before (Figure 2(c)), the GFP-tagged proteins exhibited bands at the expected molecular weight and all tested EV markers were uniformly expressed in the parental cells. The CD63-mCherry expression was relatively comparable across the samples (Figure S9(a)).

We next analysed the double-labelled EVs by western blotting. The abundance of the GFP-tagged proteins in EVs from the double-transfected samples exhibited a pattern similar to the single labelling (Figure 3(a)) with the exception of Syndecan showing a slightly fainter band at the expected size and Lamp2b displaying an extra band at around 40 kDa. As expected, no GFP was detected in controls transfected only with CD63-mCherry (N) (Figure S10 and S11). We additionally immunostained the EV samples for Lamp2b, CD81, Alix and Syntenin, which detected both endogenous and exogenous protein forms and confirmed the molecular mass displayed by GFP staining (marked with asterisks in Figure S11). As previously observed in single-labelled EVs, a truncated GFP form (~30 kDa) was faintly detected in samples from GFP-tagged Syndecan, CD81, CD9, CD63 (N), CD63 (C), Myr and SIMPLE. Probing for mCherry confirmed comparable expression of CD63-mCherry across the EV samples. The presence of the endogenous EV markers Alix, CD81, Tsg101, Syntenin was relatively similar throughout the samples, the non-EV marker Calnexin was merely present (Figure S10(a) and S11).

NTA analysis revealed a similar distribution of scatter and GFP fluorescent particles as for the single GFP labelled EVs (Figure 3(b)). Again, the tagging of tetraspanins (except CD63 with GFP in the 2^nd^ loop), Myr and Syntenin resulted in the highest number of GFP fluorescent particles (Figure S10(b)). A low level of fluorescence in the GFP channel was also detected in the EVs derived from HEK293T cells transfected with CD63-mCherry (N) plasmid only, most likely due to direct excitation of mCherry with 488 nm and imperfect spectral separation on the NTA instrument. GFP-tagged Lamp2b and SIMPLE were undetectable by NTA in fluorescence mode despite being well detectable by western blotting; CD63 (2^nd^ loop), MFGE8 (C1C2), Flotillin-2, Alix and Ubiquitin C fluorescence and expression were hardly detected with both methods.

We next evaluated the possibility of quantifying the co-expression of GFP and mCherry on dual labelled vesicles by widefield (Figure 5 and Figure S13) and confocal (Figures 5 and S12) fluorescence imaging of EVs spotted onto coverslips. To validate the approach, we first needed to exclude a possible false positive detection of co-localization due to vesicle aggregation or fusion during purification, spotting or imaging. We therefore generated separate samples of CD63-GFP and CD63-mCherry labelled vesicles from independently transfected HEK293T cells, and then mixed the two conditioned media prior to purification by UF-SEC or UC (Figure 5(a)). The diluted vesicles were spotted onto a coverslip (Figure 5(b)) and imaged by confocal microscopy. Labelled EVs were detected as light diffraction-limited GFP or mCherry fluorescent spots of uniform size, corresponding to the point spread function of the microscope optics. The colocalization was quantified based on the overlap of the point spread functions in the two fluorescent channels to derive the number of GFP, mCherry and double-positive EVs. Following UF-SEC co-purification of single-labelled EVs, no double positive spots were detected, demonstrating that the EVs remained intact and disperse after UF-SEC purification, as well as confirming the detection of single vesicles (Figure 5(c)-left). In contrast, ultracentrifugation (UC) yielded a small population of double-positive spots, revealing a certain degree of vesicle fusion and/or aggregation during the purification (Figure 5(c)-middle) that furthermore varied between experiments [49]. As positive controls, EVs labelled with a CD63-GFP-mCherry fusion protein were predominantly detected as double-positive spots (Figure 5(c)-right). Controls with EVs from either CD63-GFP or CD63-mCherry transfected cells were performed as well and confirmed exclusive detection in the correct channels (data not shown). These data validate that single vesicle imaging via confocal microscopy can be adopted to visualize single fluorescently labelled EV proteins and determine their colocalization rate at the vesicular level. Moreover, UF-SEC isolated EVs allowed to retrieve almost exclusively single vesicles, whereas caution may need to be taken with UC isolation.

Using this approach, we next determined the co-localization of the different GFP fusion constructs with CD63-mCherry. The GFP particles co-localizing with mCherry indicates the GFP fraction that is sorted into CD63-mCherry positive vesicles (% CD63mCherry vesicles carrying GFP). Likewise, the number of mCherry particles co-localizing with GFP indicates the fraction of CD63-mCherry vesicles that also carry the corresponding GFP-tagged marker (% GFP-tagged vesicles carrying CD63mCherry). As a reference, CD63-GFP showed a fraction of ca 50% of GFP vesicles that were also positive for CD63-mCherry. Interestingly, for most of the constructs that were sorted into EVs as detected by FCS and IFCM, at least 50% of the GFP-vesicles, even if low abundant, also carried CD63-mCherry. The main exception was Lamp2b which was detected predominantly in vesicles negative for CD63-mCherry, again suggesting a potential diverging vesicular trafficking biology (Figure S12).

Since widefield microscopy bears some relevant advantages over confocal imaging such as increased speed, lower photobleaching and instrumental cost, we additionally validated the single vesicle spotting and imaging protocol using high numerical NA widefield fluorescence imaging (Figure 5(d)). Detection of single EVs as light diffraction-limited spots was confirmed based on measurements of the point spread functions of the vesicles as compared to 100 nm multicolour control beads. The co-localization of all GFP-tagged reporters with CD63-mCherry tagged EVs measured by widefield fluorescent imaging (deconvolved maximum intensity projections from 5 µm z-stacks) is shown in Figure 5(e) and (f). The quantification for two constructs (Syntenin and SIMPLE) was excluded due to high background of non-vesicular GFP, resulting in deconvolution artefacts (Figure S13).

**Figure 5.**
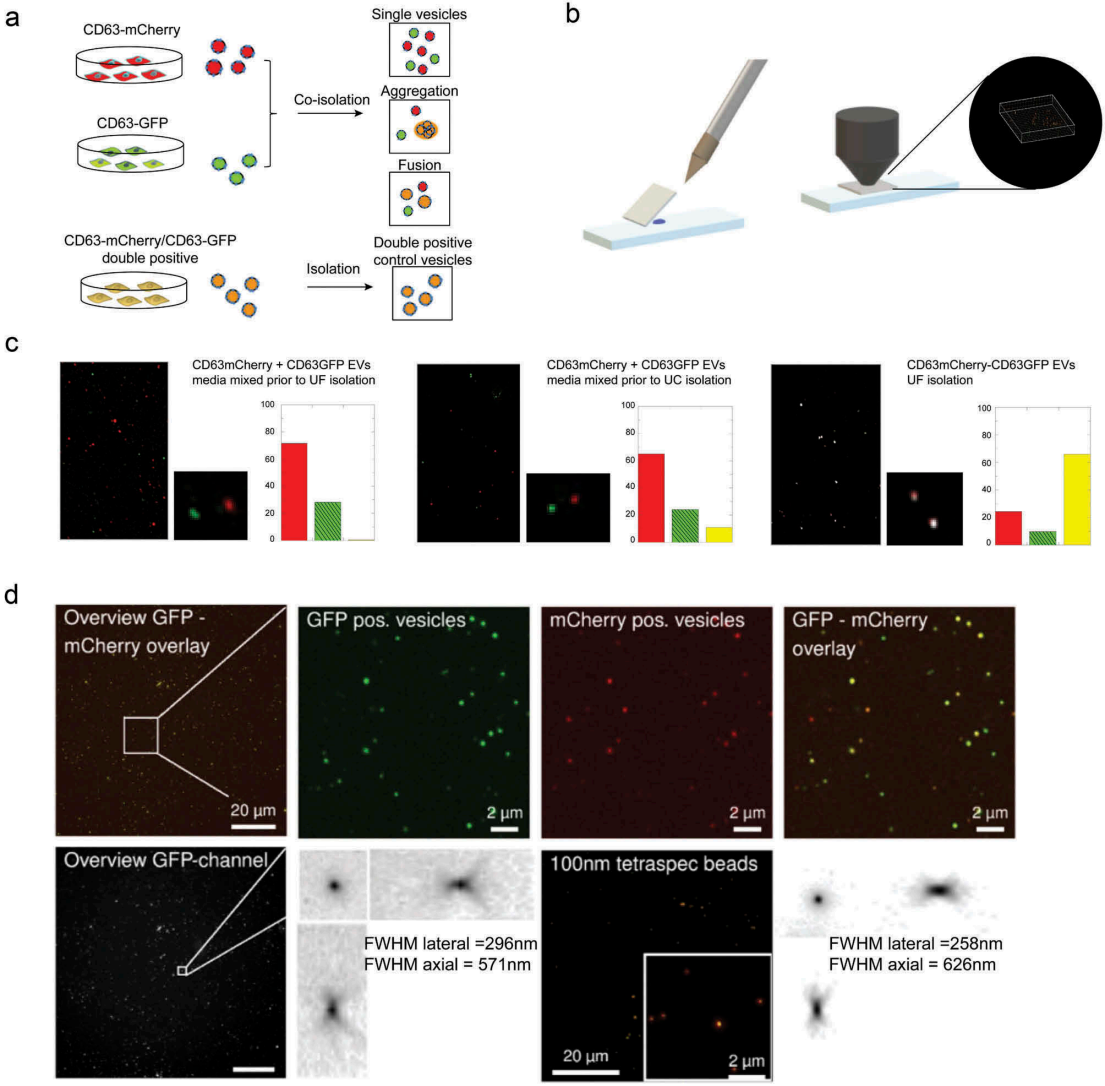
Single vesicle quantification of GFP-tagged reporters in CD63 positive vesicles. (a) HEK293T cells were transfected with either CD63-GFP or CD63-mCherry, conditioned media were mixed and EVs were co-isolated by different protocols to assess single vesicle integrity, aggregation or fusion. CD63-GFP-mCherry transfected cells were used as control. (b) 1 µl of EV sample was spotted onto glass slides, covered with 0.17 mm coverslips to generate a thin EV layer, and imaged by high NA confocal or widefield fluorescence imaging. (c) EVs from co-isolated media were detected as light diffraction-limited spots in either green or red channels with negligible occurrence of double-positive vesicles, confirming detection and integrity of single vesicles after UF-SEC (left panel). UC isolation resulted in a small fraction of double-positive vesicles (middle panel). Double labelled control EVs are shown in the right panel. The isolated EVs were analysed by confocal microscopy and the colocalization was quantified based on overlap of the point spread functions in the two fluorescent channels to derive the number of GFP (green, line pattern), mCherry (red) and GFP/mCherry (yellow) double positive vesicles. (d) CD63-GFP/mCherry double labelled control EVs imaged by high NA widefield fluorescence imaging, confirming a high degree of co-localization. The EV point spread function (PSF, bottom left) in comparison to the PSF of 100 nm Tetraspec beads (bottom right) confirms detection as light diffraction-limited spots. (e) Co-localization of different GFP-tagged EV proteins with CD63-mCherry EVs by single vesicle widefield imaging. Images are shown after deconvolution and as maximum intensity projections (MIPs from 5 µm z-stacks; red: CD63-mCherry; green: GFP. scale bar 2 µm). Co-localization quantification as indicated in the image. (f) Percentage of CD63-mCherry (red), GFP (green) and double-positive EVs (yellow) for the different GFP constructs (technical replicates shown as individual bars).

## Discussion

Sorting mechanisms and efficiencies of proteins into exosomes and EV-subpopulations are still poorly understood. In this study, we provide a systematic, single molecule-single vesicle characterization of a selection of EV-related proteins via transient overexpression of GFP-tagged proteins in parent cells. A majority of EV characterization methods and diagnostics have historically been based on “bulk” readouts. However, due to an increased awareness of the relevance of EV subpopulations, new analytical methods are focusing on single vesicle detection and analysis; multiplexed EV surface protein analysis using microfluidic system on-chip [66], single vesicle detection via flow cytometry [24,28,67] or Raman Spectroscopy with optical tweezers have been described to explore the composition of individual vesicles [68]. Here, we introduce a set of straightforward techniques to combine single vesicle analysis with single-molecule quantification. Using these methods and CD63-GFP as a reference, we provide a systematic comparison for an array of transmembrane as well as soluble and membrane-associated EV marker proteins (Table 1), with the future view of possibly replacing the fluorescent protein with therapeutic molecules or other cargo of interest.

**Table 1.** Summary of tested EV sorting domains and proteins.

Constructs		Cells	NTA		FCS				
		Main localization	Size of EVs (average mode, nm)	% GFP particles (average)	% free GFP	Average number of GFP molecules per fluorescent vesicle		Average Particle brightness (kHz)	Summary
						lowest	highest		
Transmembrane proteins	Syndecan 1-GFP (TM)	Cytosolic	128	0.83	49.96	0.7	2.1	11	Low extracellular GFP levels. mostly non-vesicular
	Lamp2b-GFP (N)	Lysosomal	114	0.34	17.32	3.3	4.4	56	Low extracellular GFP levels. moderately vesicular
	CD81-GFP (N)	Membrane	114	11.93	3.48	42.9	54.1	544	High extracellular GFP levels. mostly vesicular and bright particles
	CD9-GFP (N)	Membrane	94	19.21	4.26	9.8	46.1	658	High extracellular GFP levels. mostly vesicular and bright particles
	CD63-GFP (N)	Endosomal	109	22.4	4.46	11.3	44.1	515	High extracellular GFP levels. mostly vesicular and bright particles
	CD63-GFP (C)	Endosomal	113	32.46	7.28	10.0	62.0	181	High extracellular GFP levels. mostly vesicular and moderately bright particles
	CD63-GFP (2nd loop)	Endosomal	116	2.56	5.21	8.0	14.1	127	Low extracellular GFP levels. mostly vesicular and moderately bright particles
	mCD63-GFP (C)	Endosomal	116	22.91	4.09	37.3	42.7	615	High extracellular GFP levels. mostly vesicular and bright particles
Membrane associated proteins	MFGE8 (C1C2)-GFP (N)	Secreted	118	0.0	41.77	1.3	13.4	40	Low extracellular GFP levels. very heterogeneous population and dim particles
	Myr-GFP (C)	Membrane	114	9.5	3.81	18.5	26.5	355	Moderate extracellular GFP levels. mostly vesicular and moderately bright particles
	Flotillin 2-GFP (N)	Cytosolic	129	0.33	67.68	0.9	1.3	35	Low extracellular GFP levels. mostly non-vesicular
Soluble proteins	ALIX-GFP (C)	Cytosolic	125	0.51	32.41	1.4	1.9	84	Low extracellular GFP levels. very heterogeneous population and mostly non-vesicular
	Ubiquitin C-GFP (N)	Cytosolic	130	0.33	42.04	1.9	2.3	71	Low extracellular GFP levels. very heterogeneous population and mostly non-vesicular
	Syntenin-GFP (C)	Cytosolic	134	15.29	14.11	4.2	6.9	233	Moderate extracellular GFP levels. heterogenous population and mostly vesicular
	SIMPLE-GFP (N)	Nucleoplasmic	125	0.4	96.67	1.3	2.6	16	Low extracellular GFP levels. exclusively non-vesicular
	GFP	Cytosolic	121	2.77	22.21	1.0	40.7	166	Vesicular versus free GFP fraction and number of GFP molecules per vesicle highly variable between biological replicates

Among all EV-related target proteins selected for this screening, the tetraspanins CD9, CD81, and CD63, as well as a myristoylation domain showed the highest abundance within the EVs based on both bulk protein levels and detected numbers of GFP molecules per vesicle. Interestingly, the enrichment of the endogenous marker proteins in EVs, based on western blotting or MS proteomics, was not sufficient to ensure efficient vesicular sorting of the respective GFP-fusion protein. Likewise, the GFP-tagged proteins showed highly diverse sorting efficiencies into EVs despite similar overexpression levels in the parental cells. The most evident example is the widely used EV marker Alix which was barely detectable in EVs when overexpressed with a GFP tag. These observed differences in sorting between the endogenous and bioengineered proteins could be due to perturbation of the natural function, folding or sorting of the proteins into EVs due to the fusion of the fluorescent tag. Similarly, we observed substantial differences in loading efficiencies when fusing GFP to the N-terminus, C-terminus or second loop of CD63. This could be the result of the GFP fluorescence being quenched during the EV biogenesis; GFP sensitivity to acidic pH and local environmental changes during vesicular trafficking might have an impact on certain proteins.

We would like to point out that these data represent a comparative assessment of the eventual levels of different receptors or sorting/tagging domains obtained in EVs when expressed within parent cells from the same vector backbone, rather than an absolute molecular sorting efficiency. The latter would be an interesting question in context with EV biogenesis, which would, however, require determination of numbers of molecules sorted into EVs per total number of molecules expressed within the same parent cell(s). In this context, it has to be considered that the production of fluorescent EVs by transfected cells can lead to GFP (re-) uptake by transfected and non-transfected cells in the same well or plate, which complicates the determination of true primary expression levels. This paracrine transmission effect can be clearly seen in Figure S3. The negative – assumingly untransfected population – is being shifted to low-GFP positive as compared to the untreated sample (Figure S3(b)) after transfection with some constructs (Figure S3(c)), and not with others (Figure S3(d)). Strikingly this shift is in line with the density of GFP-tagged proteins in the EVs and is observed for Tetraspanins or Myr-GFP but not for Syndecan or Lamp2b for example. This implies that most of the cells have taken up fluorescently tagged EVs and that this effect is not negligible. This and other factors make it inaccurate to specifically quantify primary expression levels independent from secondary cell-to-cell transmission based on bulk cellular GFP levels such as by Flow Cytometry (MFI) or also western blotting and will require more sophisticated strategies.

A comprehensive analysis of CD63-GFP tagged EVs derived from transiently transfected cells, previously shown to be functionally taken up by a range of cell lines [34], confirmed largely retained composition in terms of content, morphology and physicochemical properties as compared to native vesicles. This is well in line with the fact that we detected no dramatic increase of CD63 in the EVs by MS-proteomics upon transient overexpression in the cells, resulting in an average of ~30 molecules per vesicle as determined by FCS. Due to the direct quantification of single fluorescent molecules before and after vesicle lysis and the possibility to use the identical fluorophore for virtually every fluorescent cargo as an internal reference, we propose that the quantification by Fluorescence Correlation Spectroscopy is a reliable method for single molecule-single vesicle quantification. This is inherently limited to the quantification of fluorescent molecules and does not account for the possible contribution of a fraction of non-fluorescent fusion proteins, such as due to GFP quenching by, e.g. acidic pH. With orthogonal methods emerging that have the potential of leveraging single vesicle quantification to the single molecule-single vesicle level [33], a systematic comparison with respect to the limitations of the different technologies will be warranted to approach absolute quantification in a near future.

A current unknown is the number of dye molecules required to detect, visualize and track single EVs with different fluorescent technologies, such as Fluorescent NTA, Vesicle Flow Cytometry or wide field and confocal imaging. We propose that the FCS method reported here will enable to finally address these questions. A combined assessment of the imaging data in Figure 3(d) and the FCS data in Figure 4(f) suggests that an estimate threshold for confocal imaging under standard conditions such as used here ranges at below 5–10 molecules of GFP per EV. Follow up studies shall now allow to determine these thresholds more carefully. Among the 16 constructs tested by FCS, the highest loading levels were observed for proteins of the tetraspanin family, however, none significantly exceeding the numbers of vesicular molecules observed for CD63-GFP which range at a maximum of 1.5 to 10-fold enrichment over endogenous CD63 based on semi-quantitative analysis by MS-proteomics. Whereas these numbers prove sufficient for single vesicle tracking and visualization at least in vitro, they might be a limiting factor for certain therapeutic engineering strategies depending on the function and efficacy of the payload. For instance, the decoration of EVs with targeting ligands or loading with highly active cargo such as multiple turnover enzymes or RNPs for genome engineering might be possible even with a density of a few molecules per vesicle. Also, for siRNA, typically less than 100 molecules per cell are required to induce RNA silencing [69]. Therefore, the ability to increase the number of small RNAs from one copy every 100 exosomes [70] to even one copy per vesicle should make a positive impact and allow for target knockdown upon transfer. This improvement could be achieved by stably transducing cells with the engineered protein expressing vector, leading to a more uniform and high cellular expression and subsequent increased vesicular levels.

In most of the samples, we observed a truncated form of GFP co-isolated even after size exclusion chromatography. Therefore, bulk fluorescence-based readouts as reporters for EVs need to be taken with caution. Moreover, a potential similar processing must also be considered when designing endogenously expressed therapeutics, possibly demanding for a sequence modification to mask putative cleavage sites. Particular attention should be payed when displaying cargos on the surface of the vesicles, which might cause a dissociation of the payload from the EVs. For instance, an RVG peptide tag fused to mouse Lamp2b allowed to successfully target EVs to the mouse brain [11]; however, targeting peptides fused to the N-terminus of the human Lamp2b, showed undesired proteolytic peptide degradation in other studies [71]. Upon transient Lamp2b-GFP overexpression, we observed free or truncated GFP at the cellular level but low levels in the EVs and a very weak fluorescent signal. This may be caused by quenching of the pH-sensitive fluorophore, a dissociation of the FP from Lamp2b at the cellular level prior to the vesicular sorting, or might indicate an exposure of the FP to a proteolytic environment such as in the late endosome/lysosome. All scenarios may well explain the low fluorescent signal detected with various fluorescence-based methods despite the expression of the protein at the vesicular level.

Lastly, EVs are now known to comprise a heterogenous population of vesicles that differ in size, density, content and function [48,53]. The fate of the vesicles could be determined by their cargo; therefore, uncovering the proteins that are distinctively expressed in EV subpopulations and exploit them for visualization and tracking purposes, could be crucial to unveil divergent roles of such vesicles. For this purpose, we described a straightforward method to visualize and analyse the colocalization frequency of two different fluorescently labelled proteins, that simply requires an automated fluorescent microscope. This approach revealed a substantial fraction of vesicles comprising Lamp2b-GFP devoid of CD63-mCherry, possibly indicating a divergent fate of these proteins in vesicular biogenesis. This method is further not exclusive for co-localization analysis, but can also be applied to determine vesicular aggregation as a result of the isolation procedure.

In conclusion, we provide a set of methods for straightforward quantitative and qualitative characterization of extracellular vesicles that is leveraging EV analytics to the single vesicle – single molecule level. We thereby address a long-standing gap in EV bioengineering as well as basic EV biology research for which molecular quantification is becoming increasingly essential.

## Material and methods

### Plasmids

For details on primer sequences and cloning strategies for the different constructs, refer to Table S2.

### Cell culture

For EV production, human embryonic kidney cells (HEK293T) were seeded in 15 cm culture dishes in Dulbecco’s Modified Eagle’s Medium High Glucose (DMEM, Gibco Thermo Fisher Scientific), supplemented with 10% Foetal Bovine Serum (FBS, Gibco Thermo Fisher Scientific), 20 mM L-Glutamine and 1% penicillin (100 U/ml) and streptomycin (100 μg/ml) (P/S, Sigma) and maintained at 37°C, 5% CO2 atmosphere. 7–8 × 10^6^ cells were seeded and after 24 h, were transfected with plasmids of interest complexed with branched polyethylenimine (Sigma-408727; 30 µg DNA: 45 µg PEI) in OptiMEM (Gibco, Thermo Fisher Scientific). 4 h after transfection, medium was changed to OptiMEM reduced serum medium supplemented with P/S. Cells were incubated for 48 h before EV isolation.

For other experiments HEK293T, Huh7 and B16F10 cell lines were plated in 15 cm dishes and transfected at ca 50% confluency using Lipofectamine2000 in DMEM supplemented with 10% FBS. After 4 h, cells were washed and medium was replaced with OptiMEM (GIBCO, 25 ml per dish) and cultivated for further 24 to 48h.

### Extracellular vesicle isolation

Conditioned medium (CM) from previously transfected HEK293T cells was collected and spun at 300 x g for 5 min and subsequently at 2000 x g for 10 min to remove cell debris and larger particles. The supernatant was passed through a 0.22 µm vacuum filter and subjected to different purification steps. In the experiments designed to test the sorting efficiency of the different chimeric proteins into EVs, the processed CM was concentrated by ultrafiltration (UF) with Amicon Ultra-15 100 kDa molecular weight cut-off spin-filter (Millipore) to a final volume of 1 ml and later loaded into qEVoriginal size exclusion column (Izon Science). According to the manufacturer’s instruction, the vesicular fractions were collected and further concentrated with Amicon Ultra-0.5 10 kDa molecular weight cut-off spin-filter (Millipore) to a final volume of 100 µl and stored at −80°C for further analysis.

For the other experiments, where CD63-GFP (N) was taken as a candidate, typically 100–200 ml supernatant was collected and subjected to two low speed spins 300 xg for 5 min followed by 3000 xg for 10 min to get rid of cell debris and larger particles. The supernatant was subsequently filtered through a 0.22 μm filter either using syringe filters (Merck Millipore), 50 ml Steriflip (Merck Millipore) or 250/500 ml Stericups (Merck Millipore). The pre-cleared conditioned medium was then concentrated to a volume of 0.5–1 ml on an AMICON ultrafiltration device using a 100 kDa MWCO membrane (Millipore). Enriched medium was then loaded onto a Superdex-200 column (GE Healthcare) connected to an ÄKTA prime FPLC instrument (GE healthcare) equipped with a UV flow cell. Gel filtration was performed at 4°C using sterile filtered 50 mM Tris-buffer (flow rate 0.5 ml/min). 96 individual fractions of 200 µl each were collected. NTA and FCS was performed directly in all fractions. For Western blotting, fractions were pooled (4 fractions each, omitting one fraction in between pools) and further concentrated to a volume of 30 µl on an Amicon 10 kDa MWCO spin columns (Millipore). UC isolation for the experiment in Figure 5(c) was performed following the protocol specified in Nordin et al., 2015 [49].

**Figure 5.**
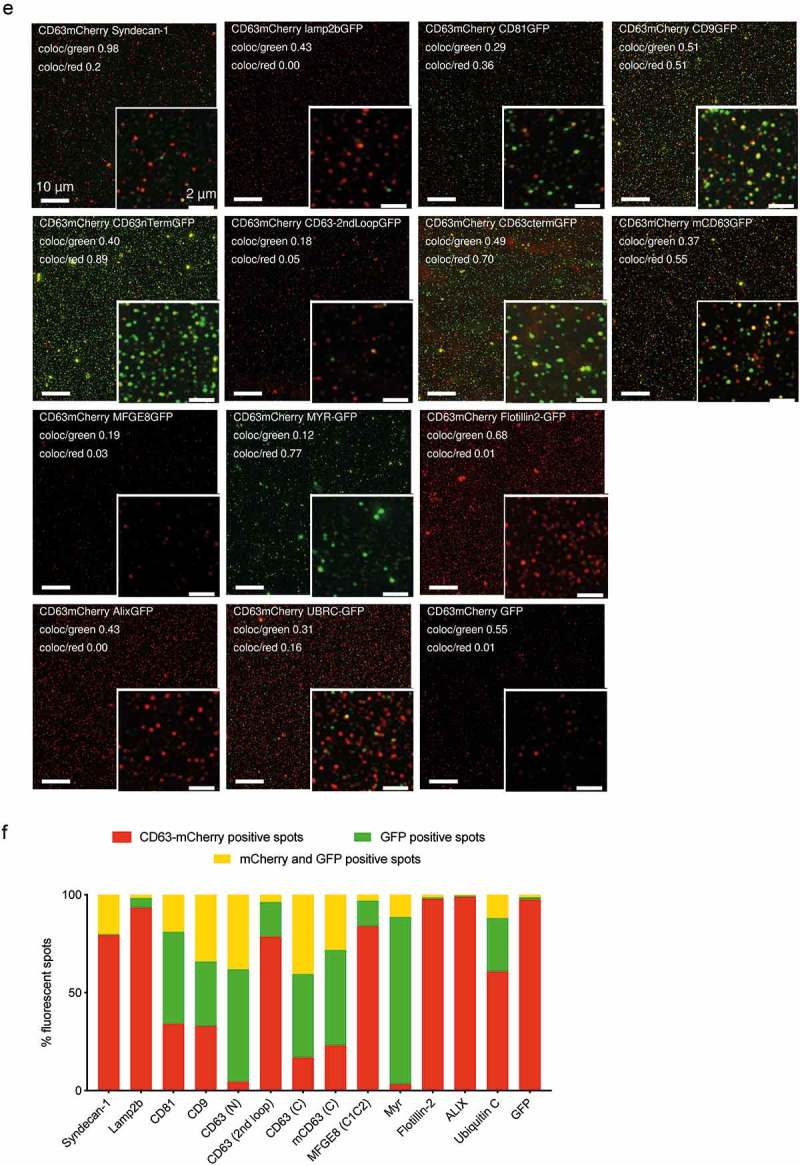
(Continued).

### Sucrose gradients

Ten 350 µl fractions with increasing sucrose density (8–80%) were overlaid via freezing between each step and topped with 12 ml conditioned, pre-cleared medium. The gradient was centrifuged for 65 h at 120,000 xg at 4°C and fractions were collected by snap freezing the gradient on dry ice and slicing into 10 or 20 fractions from the bottom. The sucrose concentration in each fraction was then determined by refractive index measurements.

### Nanoparticle tracking analysis

Particle size and concentration of the samples were determined via nanoparticle tracking analysis (NTA) using NanoSight NS500 equipped with NTA 2.3 analytical software and a 488 nm laser, as previously described [72]. Briefly, samples were diluted in 0.22 µm filtered PBS and analysed as follows: five 30-s videos were recorded per sample with a camera level of 13–14. Software settings for analysis were kept constant for every measurement (screen gain 10, detection threshold 7). For the detection of fluorescent particles, the command stage settings were changed to have a continuous flow and five 30-s videos were recorded with a camera level of 15–16. Software settings were changed to screen gain 10, detection threshold 4–5 and minimum track length to 5. Every sample was also measured in light scatter mode with a camera level of 13–14 and analysed with the same settings but detection threshold 7. The NTA measurement in flow mode were used to calculate the percentage of GFP positive particles over the total number of scatter particles in the sample.

### Western blotting

Western blotting (WB) was performed using the iBlot® system (Invitrogen, Thermo Fisher Scientific) according to the manufacturer’s instructions and as previously described [72]. Equal numbers of particles of each sample were mixed with sample buffer (0.5 M ditiothreitol (DTT), 0.4 M sodium carbonate (Na_2_CO_3_), 8% SDS and 10% glycerol) and heated at 65°C for 5 min. The mixture was then loaded onto a NuPAGE® Novex® 4–12% Bis-Tris Protein Gel (Invitrogen, Thermo Fisher Scientific) and run at 120 V in NuPAGE® MES SDS running buffer (Invitrogen, Thermo Fisher Scientific) for 2 h. The proteins on the gel were transferred to an iBlot nitrocellulose membrane (Invitrogen, Thermo Fisher Scientific) for 7 min using the iBlot system. Membranes were blocked with Odyssey blocking buffer (LI-COR) for 60 min at RT with gentle shaking. After blocking, the membrane was incubated overnight at 4°C or 1 h at RT with primary antibody solution (1:1000 dilution for anti-Alix [ab117600, Abcam], anti-Tsg101 [ab30871, Abcam], anti-Calnexin [ab22595, Abcam], anti-Syntenin [TA504796, Origene], anti-Lamp2b [ab18529, Abcam], anti-GFP [ab6556, Abcam], anti-mCherry [ab167453, Abcam]; 1:200 dilution for anti-CD81 [sc-9158, Santa Cruz Biotechnology] and 1:10,000 dilution for β-actin [A5441, Sigma]). The membrane was washed with PBS supplemented with 0.1% Tween 20 (PBS-T) for 5 min, 5 times and incubated with the corresponding secondary antibody (LI-COR) for 1 h at RT (1:15,000 anti-mouse IRDye®800CW or 680LT to detect Alix, Syntenin, β-actin; 1:15,000 dilution anti-rabbit IRDye®800CW or 680LT to detect CD81, Tsg101, Calnexin, Lamp2b, GFP and mCherry). Membranes were washed with PBS-T 5 times within 25 min, one time with PBS and visualized on the Odyssey infrared imaging system (LI-COR).

For other experiments where aliquots of sucrose gradient fractions or pooled and spin column concentrated gel filtration fractions were used, samples were heated in SDS sample buffer for 10 min at 70°C and electrophoresed on 4–12% NuPage gels (Life Technologies). Proteins were transferred to Protran nitrocellulose membranes (Whatman) using a SD Transblot system (BioRad) and blocked for 1 h in PBS with 5% (w/v) milk powder (BioRad) at RT prior to primary antibody incubation either for 2 h in blocking buffer at RT or overnight at 4°C. Immune complexes were visualized using HRP-conjugated secondary antibodies (Santa Cruz) on a Biorad XRS system. Antibodies used: CD63 (sc-15363, Santa Cruz, 1:500); PDC6I/Alix (ab117600, Abcam, 1:500 dilution), Tsg101 (ab83, Abcam, 1:500 dilution); Lamp2 (ab25631, Abcam, 1:500 dilution); Calnexin (ab22595, Abcam, 1:10,000 dilution); GFP (ab290, Abcam, 1:1000 dilution); Calreticulin (A301-130A, Bethyl laboratories, 1:10,000 dilution);

### Cryo-transmission electron microscopy (cryo-tem)

A 4 µl aliquot of each sample was adsorbed onto glow-discharged holey carbon-coated grid (Quantifoil, Germany), blotted with Whatman filter paper and vitrified into liquid ethane at −178°C using a Vitrobot (FEI, Eindhoven, Netherlands). Frozen grids were transferred onto a Philips CM200-FEG electron microscope (FEI, Eindhoven, Netherlands) using a Gatan 626 cryo-holder (GATAN Inc, Pleasanton, USA). Electron micrographs were recorded at an accelerating voltage of 200 kV and a nominal magnification of 50ʹ000x, using a low-dose system (10 e−/Å2) and keeping the sample at −175°C. Defocus values were ranging from -2 µm to −3 µm. Micrographs were recorded at 4K × 4K with a CMOS camera (TVIPS, Germany).

### Fluorescence correlation spectroscopy (FCS)

EVs quantification and characterization via FCS was essentially performed as described elsewhere [49]. Briefly, samples were measured on a Clarina II Reader (Evotec Technologies) with 488 nm argon ion laser excitation, a 40x water immersion 1.15 N.A. objective (UAPO Olympus), 50 micrometre pinhole and a SPCM-AQR-13FC avalanche photodiode (Perkin-Elmer Optoelectronics). The confocal volume was calculated in approximation according to [73] using the measured diffusional correlation time t_diff_ of free dye (Alexa488, Life Technologies), the known translational diffusion coefficient of Alexa488 (Molecular Probes; D = 280mm^2^/s) and the axis ratio fitted from calibration measurements. For each sample, several dilutions were made and measured in a 96-well glass bottom plate (Whatman) with 30 repetitive measurements of 10 s each. NP40s at 1% v/v (Nonidet P40 substitute, G-Biosciences) was used to induce vesicle disruption for determination of detergent sensitivity and quantification of GFP molecules per vesicle. For testing of different treatments for vesicle disruption, CD63-GFP HEK293T EVs isolated by UF-SEC were incubated with different additives or under different conditions as indicated, allowed to adjust to room temperature as indicated and measured by FCS as above. The fraction of intact vesicles was determined based on a two-component fit, setting the translational diffusion time of non-vesicular GFP to the values determined by a 1-component fit in presence of 1% NP40s.

### Fusion proteins expression levels in HEK293T by flow cytometry

HEK293T were seeded, cultured and transfected as mentioned above. 48 h after transfection cells were trypsinized, collected and transferred to 5 ml round bottom polystyrene tubes (Corning). The cells were spun down at 900 xg for 5 min, the pellet was washed once with cold PBS and resuspended in 0.5 ml of PBS containing 1 mM EDTA and 2% FBS (Gibco, Thermo Fisher Scientific). 4ʹ,6-diamidino-2-phenylindole (DAPI) staining was added to all samples to exclude dead cells from the analysis and doublets were excluded by forward/side scatter area versus height gating. The samples were measured with MACSQuant Analyser 10 cytometer (Miltenyi Biotec) and data was analysed with FlowJo software (FlowJo, LLC, version 10.0.7).

### Liquid chromatography tandem mass spectrometry (LC-MS/MS) of HEK293T derived EVs

LC-MS/MS analysis on UF-SEC isolated HEK293T-EVs (Figure 2(a)) was performed as previously described [49].

Proteomic analysis depicted in Figure 1(f) was performed on UF-SEC isolated EVs separated by SDS-PAGE on a NuPAGE 4–12% (Life Technologies) gel and stained with a Colloidal Coomassie stain (Sigma). Sixteen equal sized slices were excised from each of the gel lanes. In-gel digestion and subsequent identification by liquid chromatography coupled with tandem mass spectrometry was performed as previously described [74], with the exception that a mix of Trypsin and Endopeptidase Lys-C (Promega) was used instead of trypsin alone. Database searches were done with Mascot (version 2.4, Matrix Science) against the UniProt database (release of April 2013) concatenated with a reversed version and supplemented with known contaminants (such as trypsin, BSA and commonly used tags). Protein identifications were validated and summarized in Scaffold (version 4.0.3, Proteome Software Inc.), setting the protein identification threshold at a 1% false discovery rate (FDR) in the reversed database. At these settings, peptide FDR was 0.05%. The resulting protein list is provided as Table S1. Keratin contaminants were removed and are listed separately. Trypsin and Lys-C were also removed from the list. Total spectral count is provided as a semi-quantitative measure, as well as the number of unique peptides for each protein (Table S1). The spectral count is shown without correction for the total number of assigned spectra in each sample (24,626 for GFP-CD63 and 21,234 for the untransfected sample).

### Single and double vesicle imaging (spotted vesicles)

EVs from single and double transfected HEK293T cells were imaged by confocal fluorescence microscopy (Zeiss LSM700) using 63x magnification and numerical aperture 1.4 or widefield with a Plan Apo 60x Na1.42 plus optovar 1.6x (API DeltaVision) on Photometrics CoolSNAP HQ2, interline transfer CCD (pixel size 6.45 µm), after spotting 1 µl onto a glass slide and covering the sample with a #1.5 cover slip.

For the widefield imaging, a 5 µm dual channel fluorescence stack (GFP & mCherry Chroma filter-set) with 130 nm steps was acquired and deconvolved with the DeltaVision software (Enhanced Ratio -aggressive- algorithm with 10 cycles) fed with a measured 0.2 µm bead point spread function (OTF).

Vesicles were detected as light diffraction-limited GFP or mCherry fluorescent spots of uniform size corresponding to the point spread function of the microscope, confirming recovery of single vesicles. For the quantification deconvolved stacks were processed to maximum intensity projection images (MIP- Delta vision software) and a colocalization channel was generated (Bitplane- Imaris 9.3) with an intensity threshold of 300 au (>2 fold camera background intensity ~130 au). Then spots in the green-, the red- and the coloc-channel were quantified with the spot detector wizard of the Imaris software with a minimum diameter of 0.2 µm and a quality threshold of 500 au (intensity in centre of spot). The CD63mCherry SIMPLE-GFP & CD63mCherry Syntenin sample had high green fluorescent background. It was not possible to image and analyse the sample with identical settings as all other samples. Therefore, a comparable quantification of these two samples was not possible.

### Imaging flow cytometry

The GFP-tagged EVs were analysed using an ImageStreamX MkII instrument (ISX; Amnis/MilliporeSigma) equipped with 5 lasers (70 mW 375 nm, 100 mW 488 nm, 200 mW 561 nm, 150 mW 642 nm, 70 mW 785 nm (SSC)) as previously described [33]. All analyses were performed by using the 60x objective (Numerical aperture: 0.9, Depth of Field: 2.5 µm) and deactivated Remove Beads option. All lasers were set to maximum powers, and all data was acquired with a 7 µm core size and low flow rate at around 0.38 µl/min. GFP signals were collected in channel 2 (480–560 nm filter). Channels 1 (430–480 nm filter) and 9 (570–595 nm filter) were used as brightfield channels and channel 6 (745–800 nm filter) for SSC detection. Dulbecco’s PBS pH 7.4 (Gibco) was used without further filtration as sheath fluid. Data was analysed with optimized masking settings as described before using Amnis IDEAS software (version 6.2.64.0) and FlowJo v. 10.5.3 (FlowJo, LLC).
