# Supplementary material for: Systematic characterization of extracellular vesicle sorting domains and quantification at the single molecule – single vesicle level by fluorescence correlation spectroscopy and single particle imaging
Source: J Extracell Vesicles. 2019 Sep 18;8(1):1663043. doi: 10.1080/20013078.2019.1663043 (PMC6758720; doi:10.1080/20013078.2019.1663043)
Supplement: Supplemental Material [file ZJEV_A_1663043_SM7244.zip › ZJEV_A_1663043_Supplementary/Corso_Heusermann et al._Supplementary Figures.1.docx]

Supplementary Information

**Systematic characterisation of extracellular vesicles sorting domains and quantification at the single molecule – single vesicle level by fluorescence correlation spectroscopy and single particle imaging**

Giulia Corso*^1^, Wolf Heusermann*^2,3^, Dominic Trojer^2^, André Görgens^1,4^, Emmanuelle Steib^2,5^, Johannes Voshol^2^, Alexandra Graff^6^, Christel Genoud^6^, Yi Lee^1,7^, Justin Hean^2^, Joel Z. Nordin^1^, Oscar P.B. Wiklander^1^, Samir EL Andaloussi^1+^, Nicole Meisner-Kober^2,8+^

Affiliations:

1. Department of Laboratory Medicine, Karolinska Institutet, Stockholm, Sweden
2. Novartis Institutes for Biomedical Research, Basel, Switzerland
3. Current affiliation: Imaging Core Facility, Biozentrum, University of Basel, Switzerland
4. Institute for Transfusion Medicine, University Hospital Essen, Essen, Germany
5. Current affiliation: Department of Cell Biology, Sciences III, University of Geneva, Geneva 1211, Switzerland
6. Friedrich Miescher Institute for Biomedical Research, Basel, Switzerland
7. Current affiliation: A*star Genome Institute of Singapore, Singapore
8. Current affiliation: Department of Biosciences, University of Salzburg, Salzburg, Austria

*^*These authors contributed equally^*

*^+Corresponding authors^*


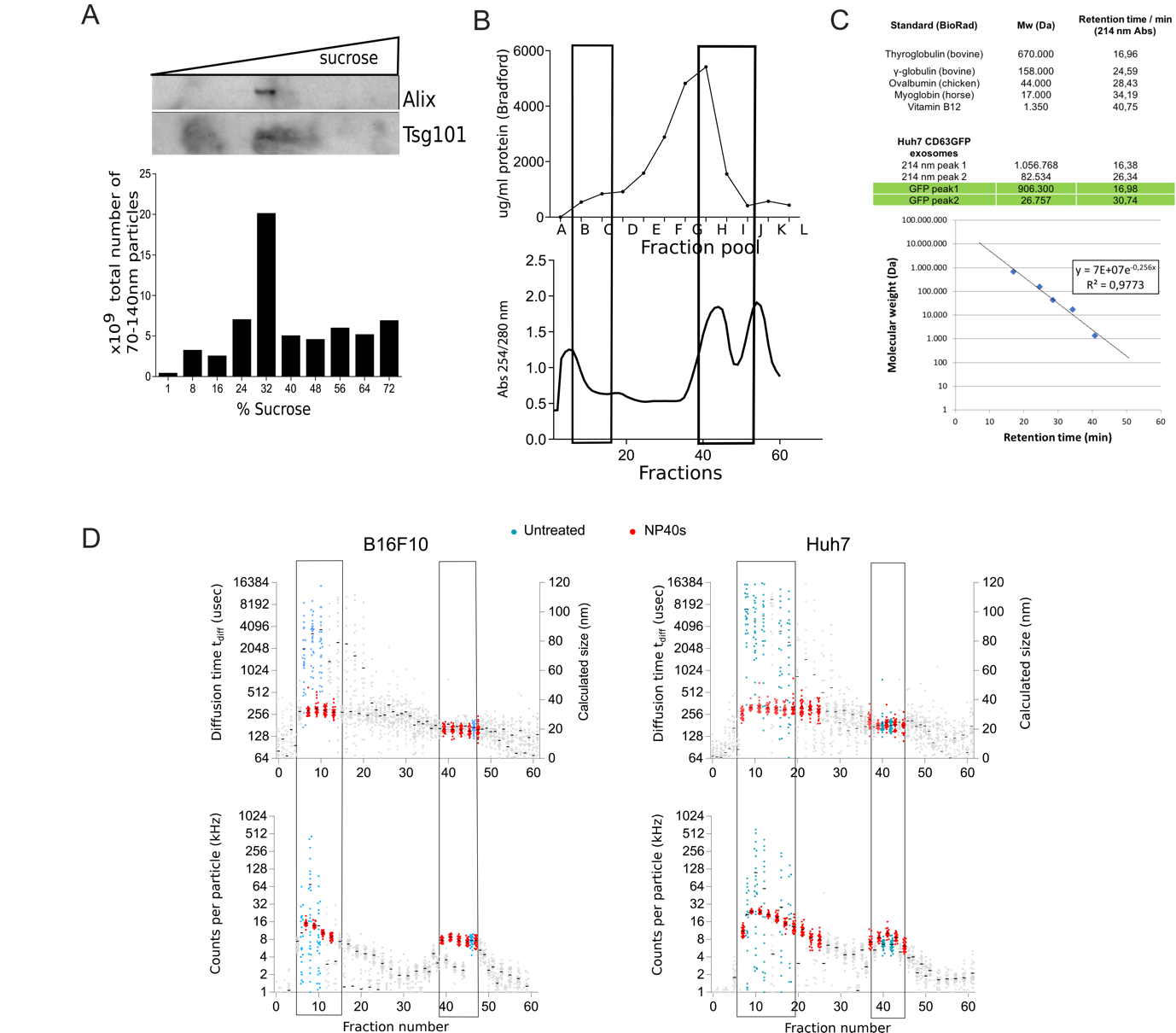


**Supplementary Figure 1. Characterization of CD63-GFP labelled EVs. (A)** Conditioned medium from untransfected HEK293 cells was directly fractionated on a sucrose sedimentation gradient without any prior ultracentrifugation step**.** X-axis shows measured sucrose concentrations in the collected fractions. Particles with a size of 70‑140 nm (as measured by NTA) co-fractionate with the EV markers Alix and Tsg101, peaking at ca 32-36 % sucrose. **(B)** Fractions from SEC of CD63-GFP HEK293 exosomes shown in Figure 1c were analyzed for total protein concentration by a Bradford assay (upper panel). The ratio between the 254/280 traces is shown in the lower panel. **(C)** Retention time analysis of SEC of exosome samples using a size standard. Upper size separation limit of the column: 600 kDa. Exclusion limit: 1.3 MDa. **(D)** Individual fractions from SEC of B16F10 CD63-GFP and Huh7 CD63-GFP EVs were analyzed by FCS with (red data points) and without (blue data points) vesicle disruption by the detergent NP40s. The translational diffusion time (tdiff, upper panel) and molecular brightness (CPP, second panel) are depicted across the fraction number from the SEC. Frames indicate fractions comprising the two main fluorescent populations.

**
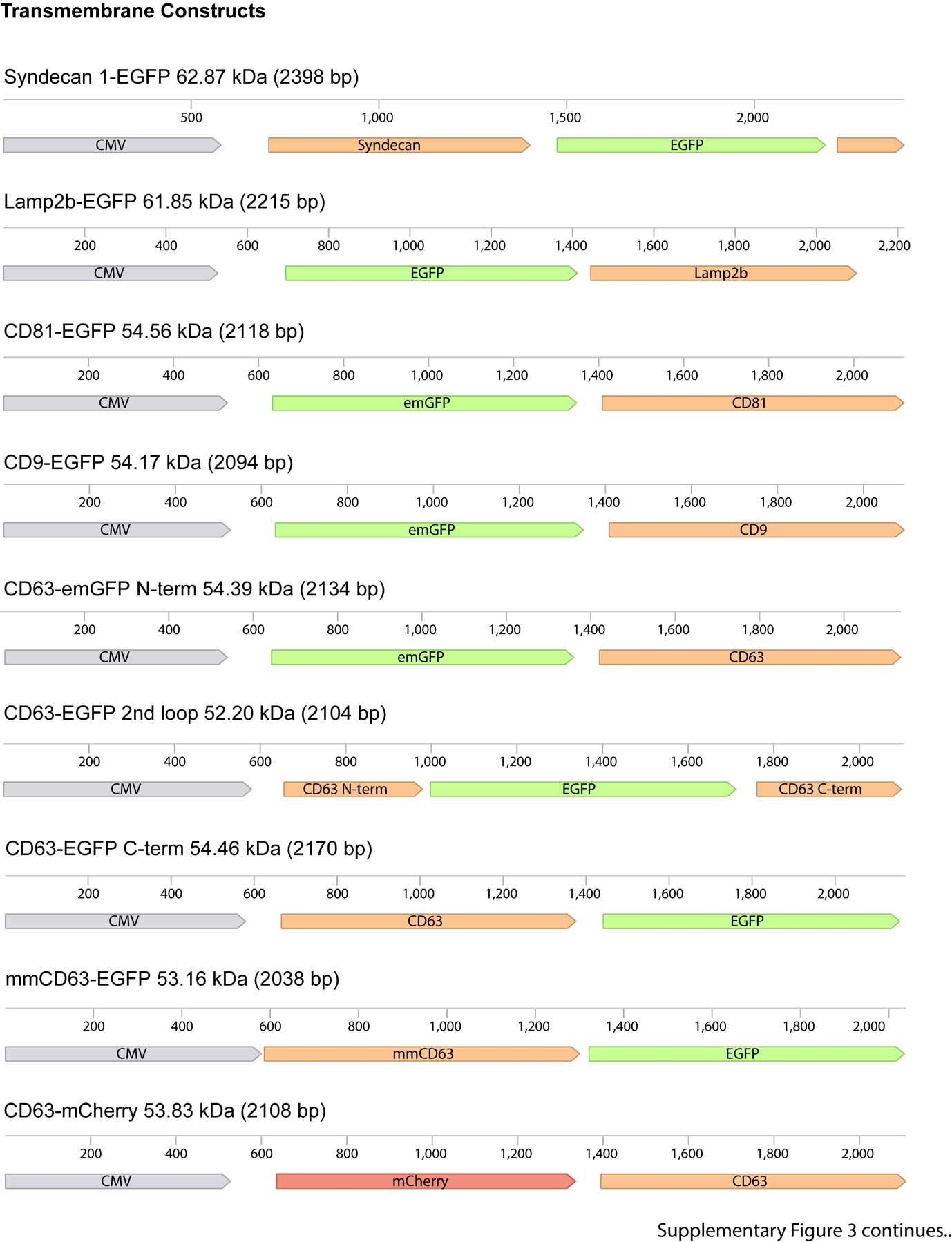
**

Supplementary Figure 2 continues in the next page..

**
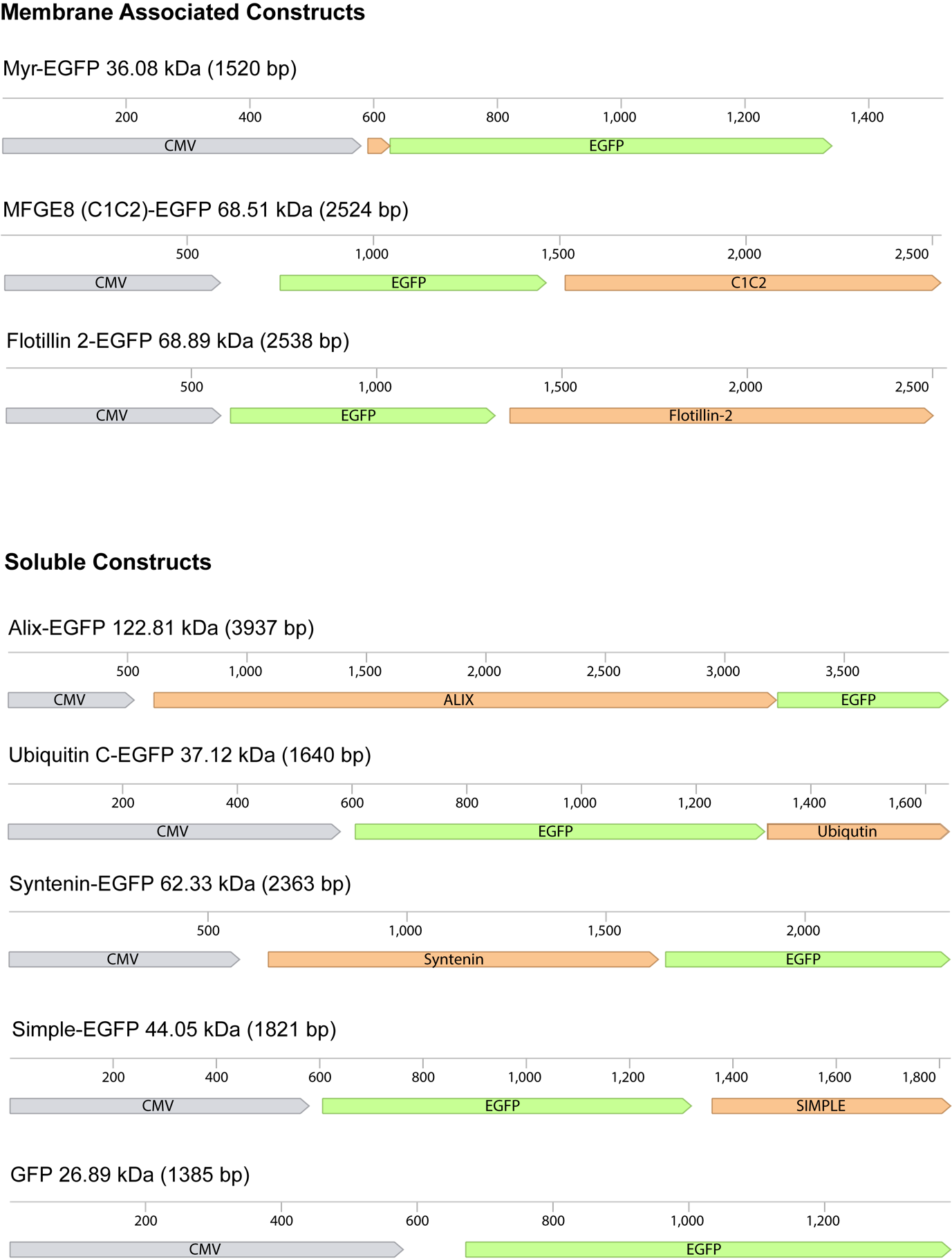
**

**Supplementary Figure 2. Graphical representation of the GFP fusion constructs.** Cytomegalovirus (CMV) promoter (grey), enhanced green fluorescent protein (EGFP, in green), emerald green fluorescent protein (emGFP, in green), mCherry fluorescent protein (mCherry, red) and DNA sequences encoding for the different EV sorting proteins (orange). For each construct, the molecular weight and the nucleotide length are specified.

**
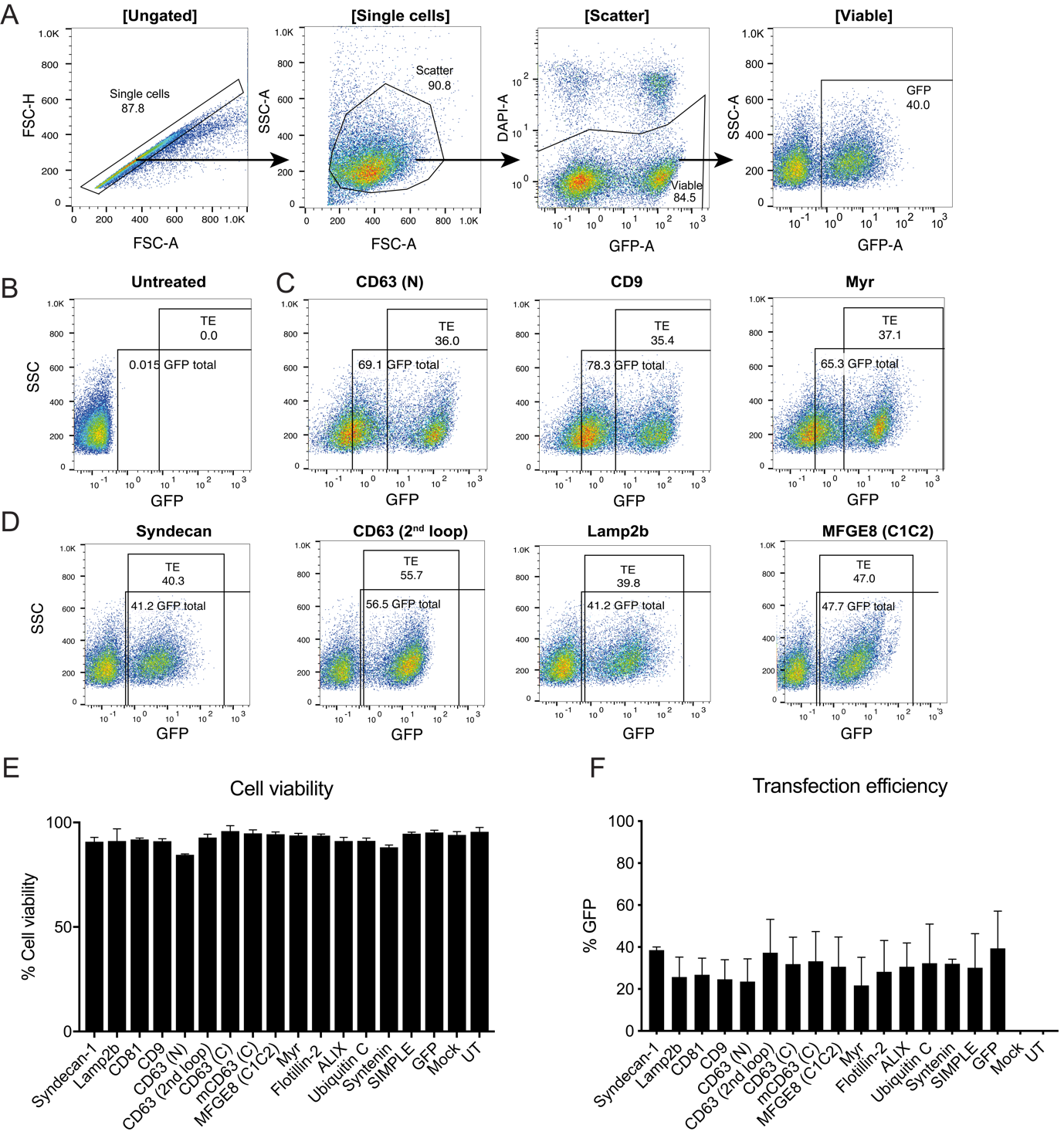
**

**Supplementary Figure 3. Flow cytometry on transfected HEK293T cells.** Plasmids encoding for 16 different GFP-fused proteins were complexed with PEI and transfected on to HEK293T cells. 48h later, transfected HEK293T cells were analyzed by flow cytometry to quantify the cell viability **(A, E)** using DAPI staining compared to non-transfected cells (UT; N=3) and transfection efficiency **(A, B, C, D, F)**. **(A)** Gating strategy applied to identify single cells, and quantify viability (DAPI-negative) and transfection efficiency (%GFP). Plots in **(B-D)** show examples for the quantification of transfection efficiencies (TE) accounting for potential EV-mediated shifts of the respective negative cell population appearing as GFP positive, potentially due to uptake of efficiently GFP-tagged EVs. Examples show UT cells **(B)**, cells transfected with CD63(N), CD9, Myr, where the population shift is more prominent **(C)** and Syndecan, CD63 (2^nd^ loop), Lamp2b, MFGE8 (C1C2) where there is no shift **(D)**. **(F)** The fluorescence intensity was measured in each sample by flow cytometry. The transfection efficiency is expressed as the percentage of gated fluorescent cells (TE) of viable gated events as defined in (A). N=3.

**
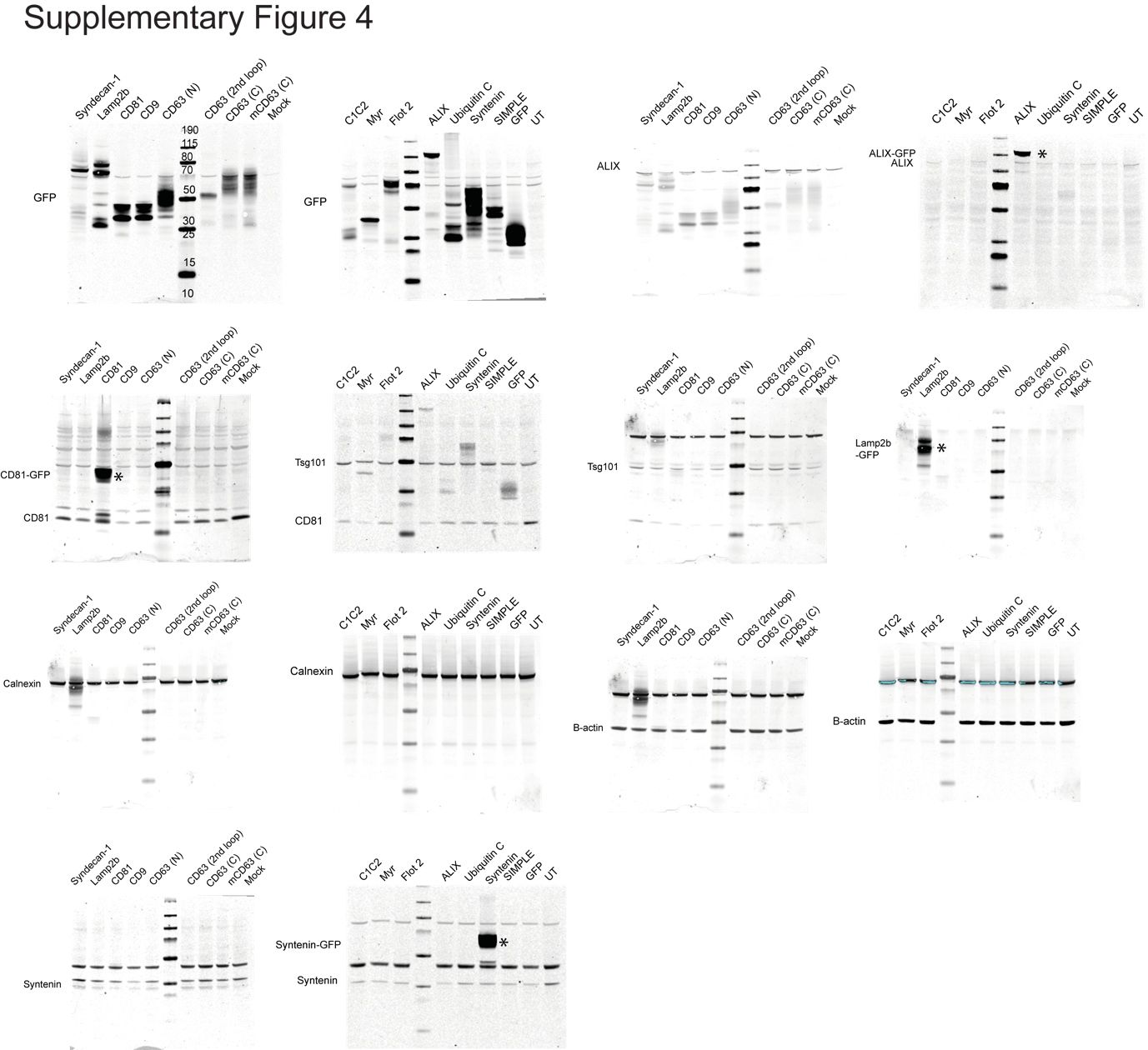
**

**Supplementary Figure 4.** Uncropped Western Blot analysis of Figure 2C.


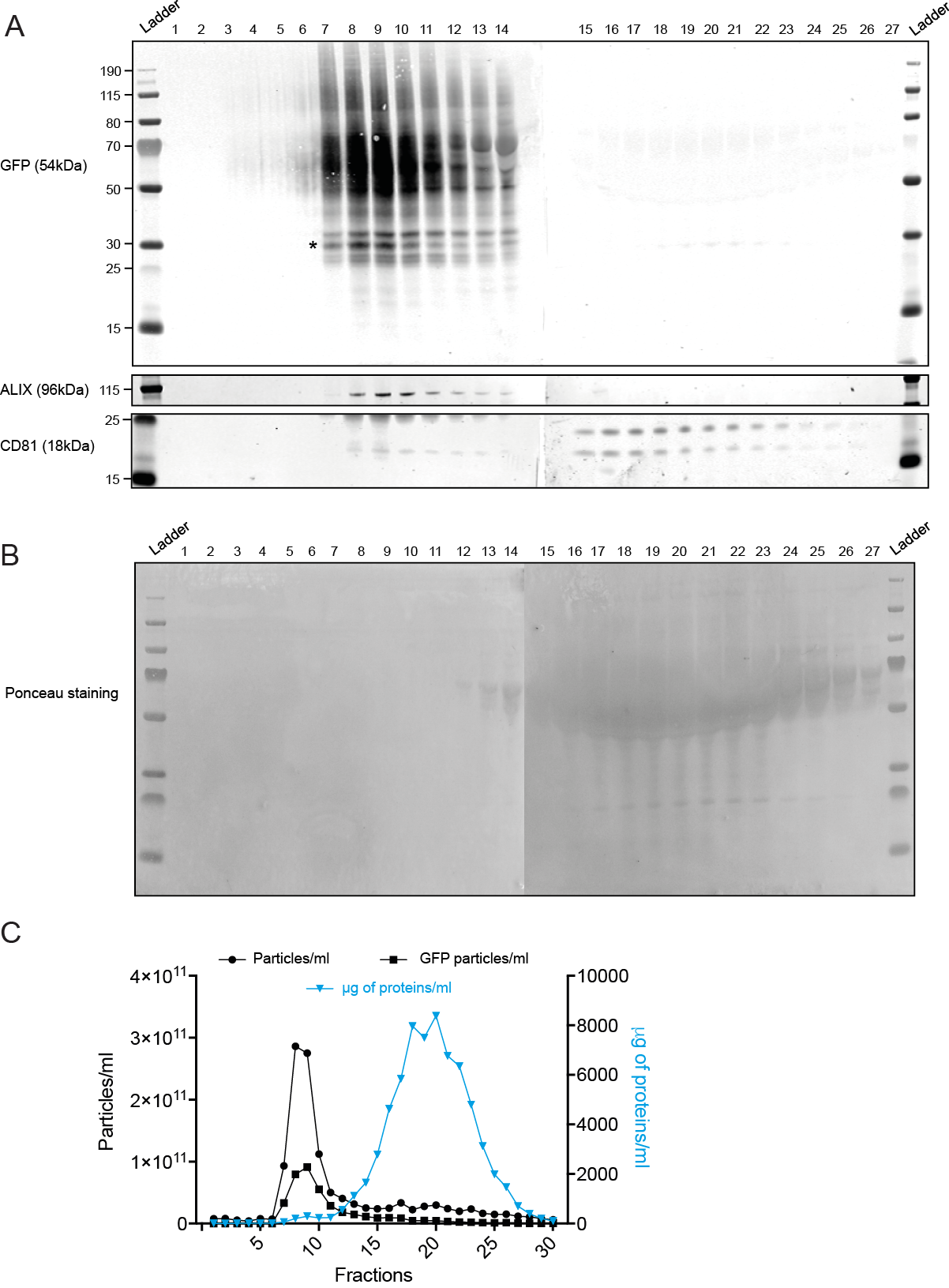


**Supplementary Figure 5. (A)** UF-qEV fractionation of HEK293T:CD63-GFP derived EVs analyzed by Western Blot. The presence of CD63-GFP in the vesicles was detected with an anti-GFP antibody (truncated GFP is indicated by an asterisk) whereas the expression of EV markers was identified via anti-Alix and anti-CD81 antibodies. **(B)** Total protein staining with Ponceau S of UF-qEV fractions. **(C)** Individual fractions were analyzed by NTA for total (black dot) and fluorescent (black square) particles concentration and by DC protein assay for protein concentration (blue triangle; right Y-axis).

**
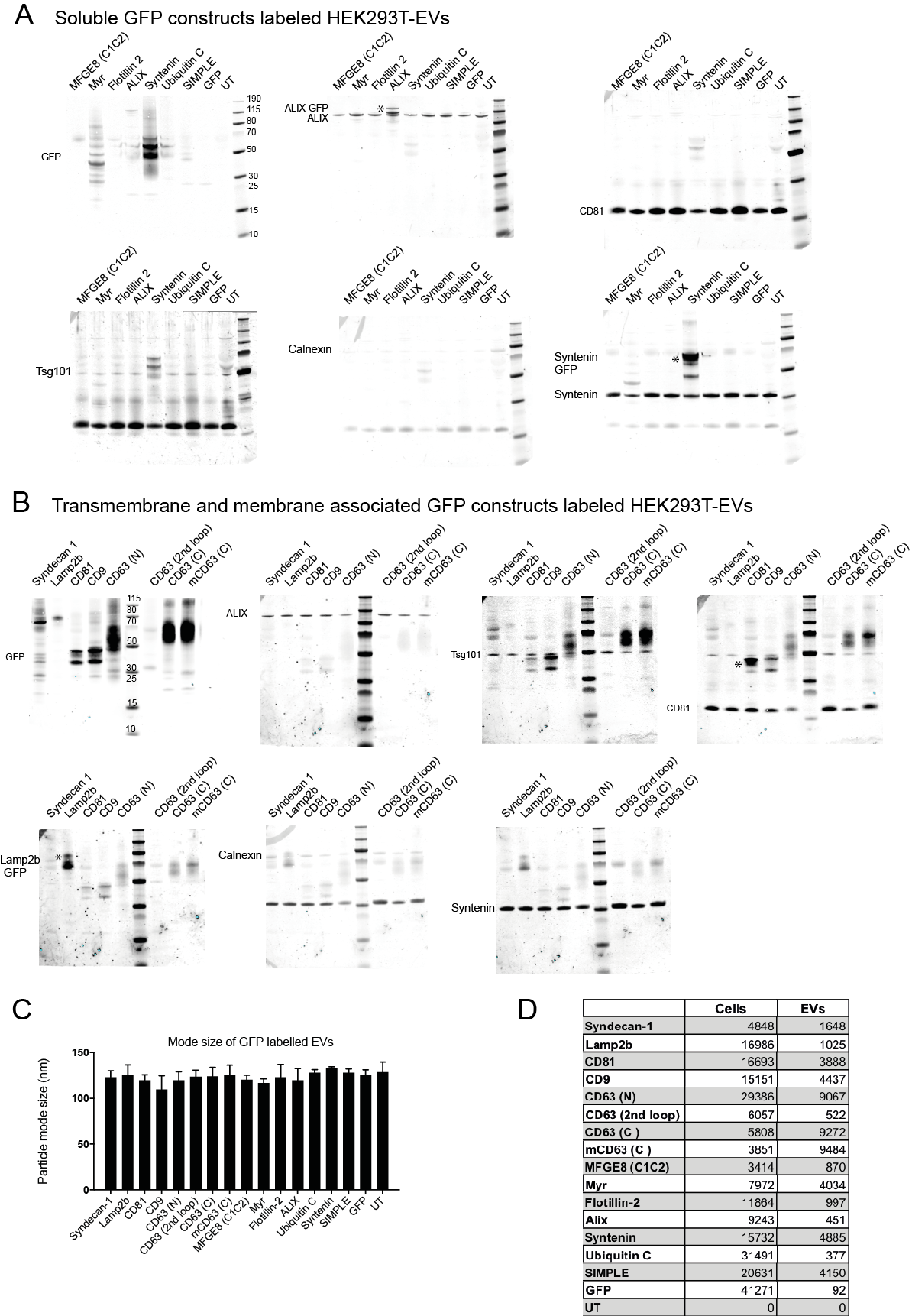
**

**Supplementary Figure 6.** Uncropped Western Blot analysis of Figure 3A for **(A)** soluble GFP-constructs**, (B)** transmembrane and membrane associated ones. Engineered protein form indicated with asterisks. **(C)** Mode size of isolated particles measured by NTA. (N=3) **(D)** WB quantification of GFP expression in single transfected HEK293T cells (from Figure 2C) and EVs (from Figure 3A). The values depicted in the table correspond to the area under the curve, equivalent to the intensity of each band. Only the bands corresponding to the expected size of each GFP-tagged construct were analysed. The quantification has been performed using the ImageJ software v.2.0.0.

**
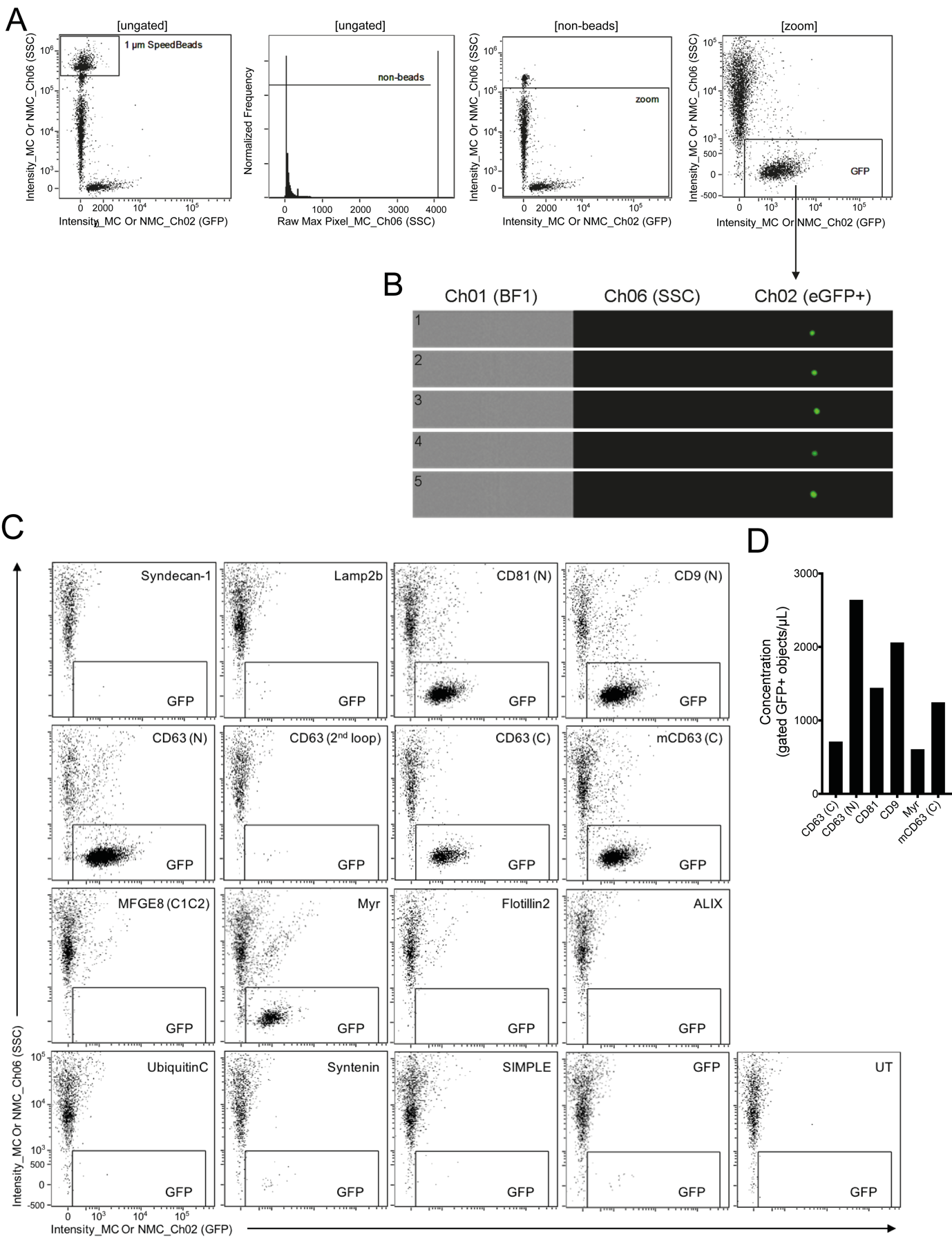
**

**Supplementary Figure 7. Imaging flow cytometry (IFCM) of different GFP tagged EVs (A)** Applied gating strategy for IFCM analysis of GFP EVs. **(B)** Example event images for GFP+ gated single green fluorescent EVs without detectable signals in the Brightfield (BF1, Channel 1) and SSC (Channel 6) Channels. **(C)** Scatter plots of the different EV domain-GFP tagged EVs analysed by imaging flow cytometry (Amnis ImageStreamX MkII instrument). The rectangle area depicts the gating for GFP positive EVs monitored in each sample. Plots shown are not pre-gated, and 1 µm speedbeads scattering at higher SSC values than 10^5^ were not acquired. All samples were measured from CM without further dilution, data was recorded for 5 minutes per measurements, **(D)** concentration values reflect the mean out of 3 replicates with highly similar outcome.

**
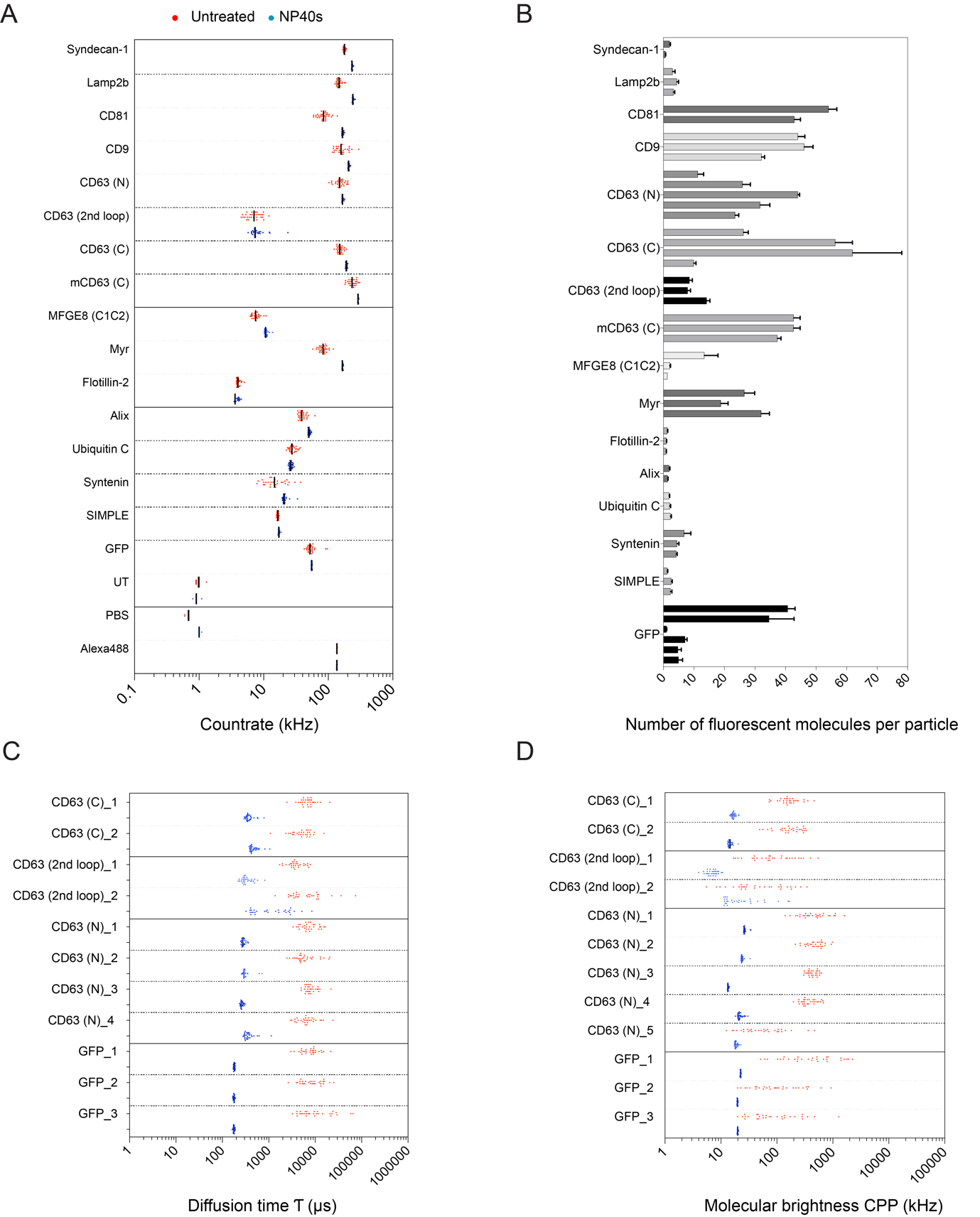
**

**Supplementary Figure 8. (A)** GFP tagged EVs analysed by FCS depicting the countrate (kHz). **(B)** Number of fluorescent molecules per particle from several independent transfections and isolations. (**C)** Diffusion time and **(D)** molecular brightness CPP measured by FCS in few of the EV replicates expressing soluble GFP and GFP fused to different regions of CD63.


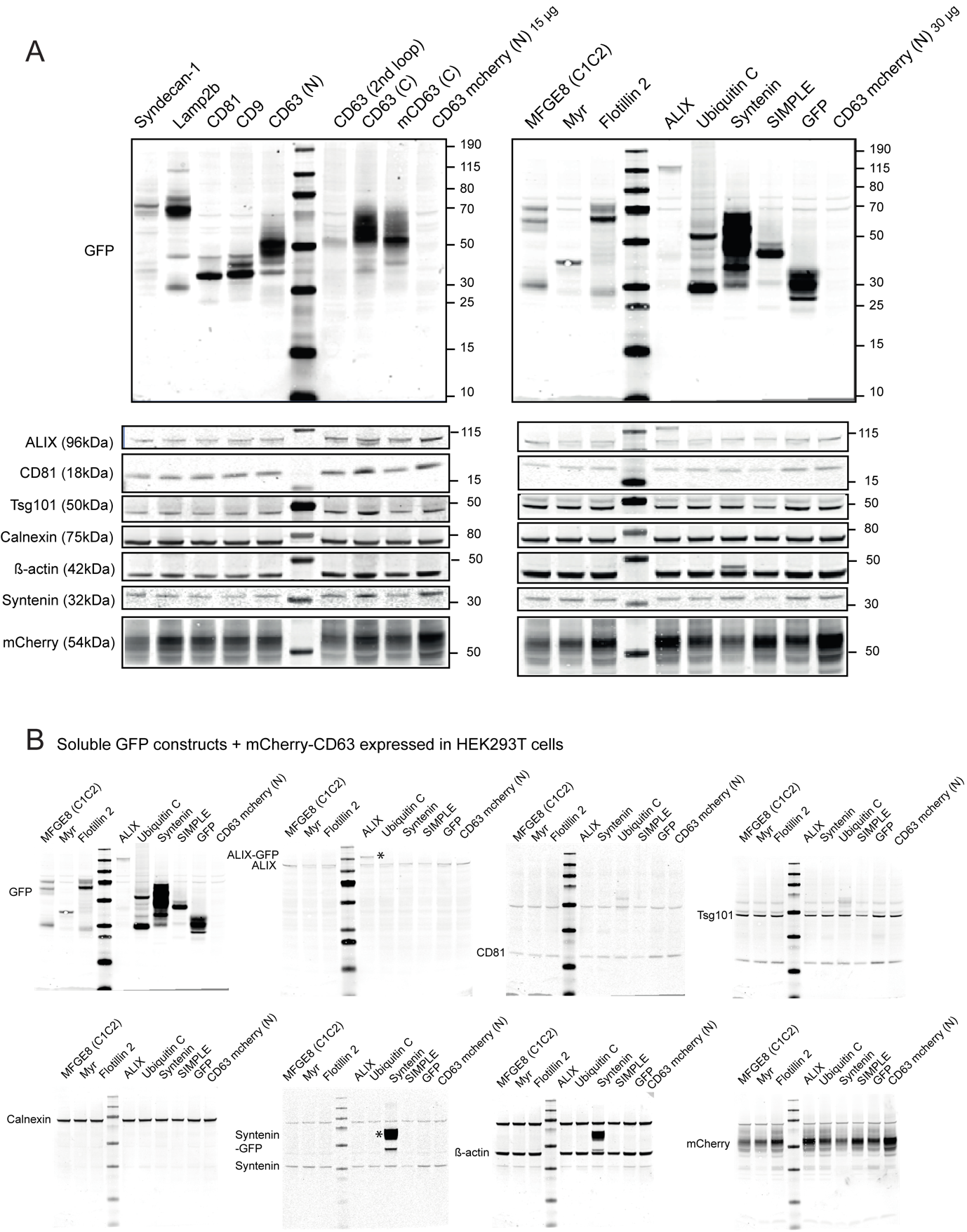


Supplementary Figure 9 continues in the next page..


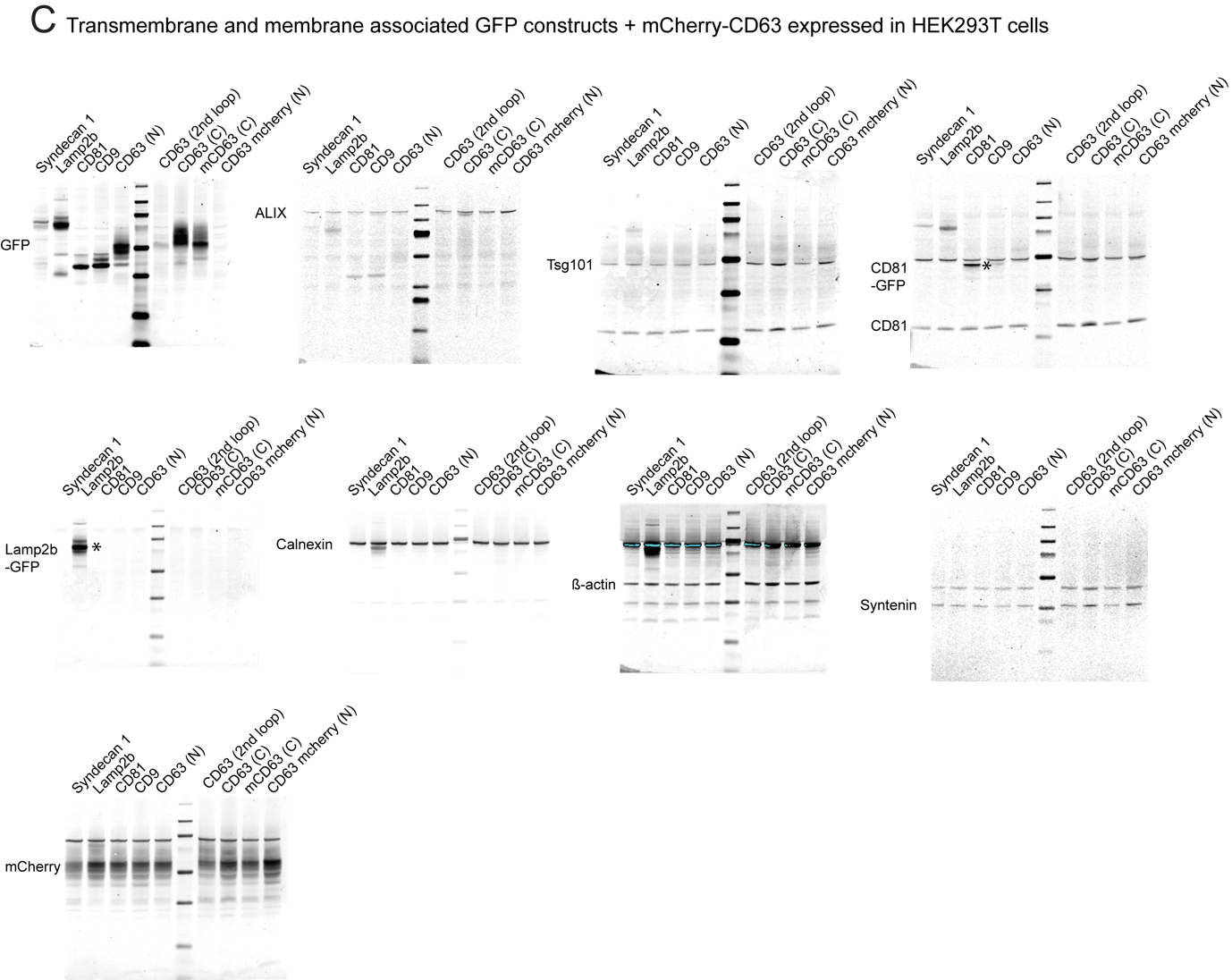


**Supplementary Figure 9. (A)**  Western Blot analysis of double transfected HEK293T cells. Uncropped WB images of **(B)** soluble, **(C)** transmembrane and membrane associated GFP-constructs transfected in combination with CD63-mCherry into HEK293T cells.


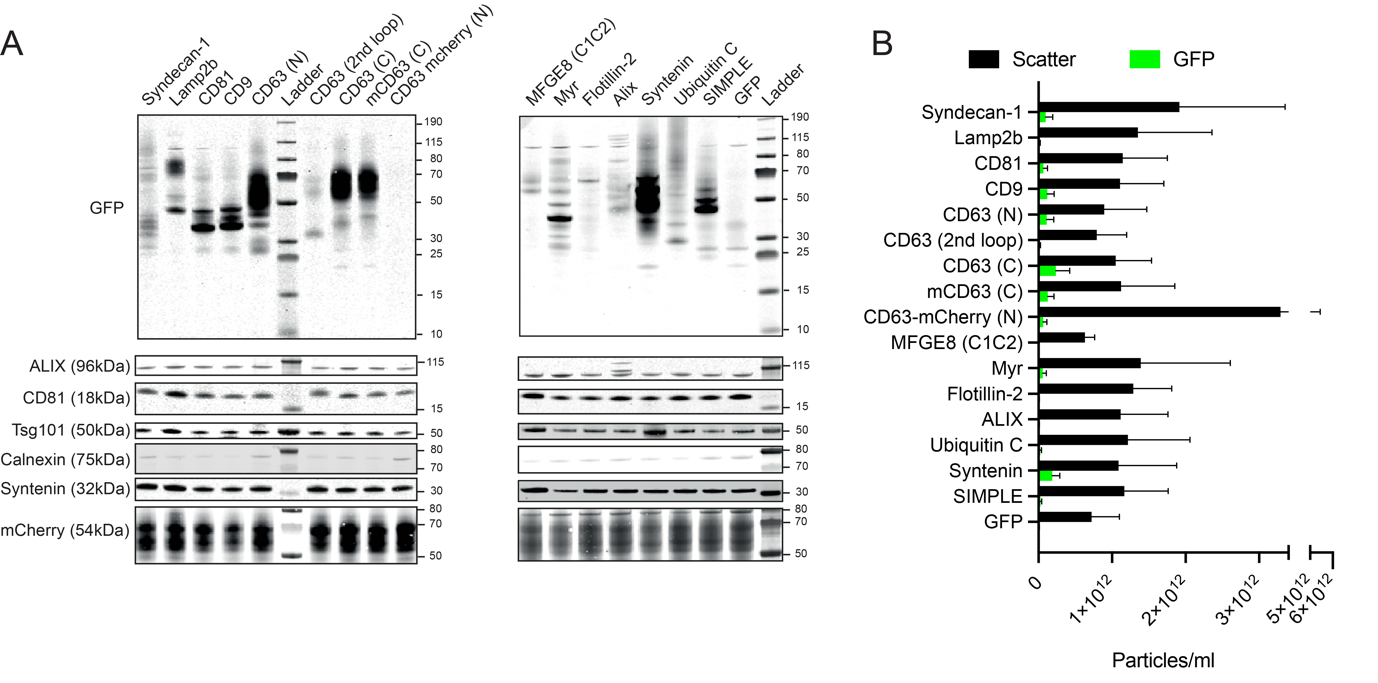
 **Supplementary Figure 10. (A)** Western blot illustrating the molecular weight (kDa) of each sorted GFP-fused protein (upper panels) in EVs co-expressing CD63-mCherry (lower panels) and the expression of several EV markers across the samples (lower panels). **(B)** NTA analysis of isolated EVs showing the total particle concentration (black) and the concentration of GFP positive particles (green) in each double labelled EV sample (N=3).


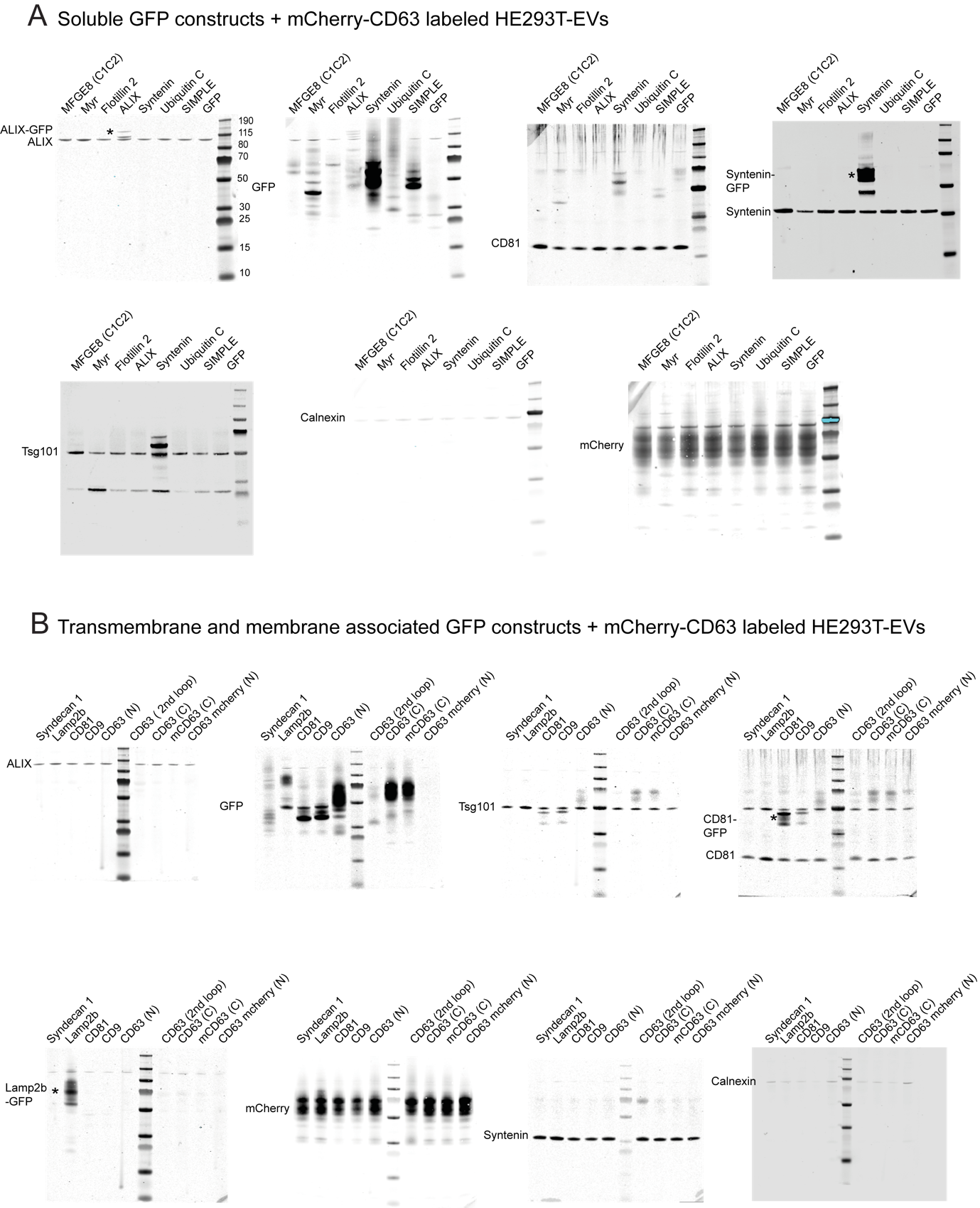


**Supplementary Figure 11.** Uncropped Western Blot analysis of Supplementary Figure 10 for **(A)** soluble GFP-constructs and **(B)** transmembrane and membrane associated ones in combination with CD63-mCherry. Engineered protein form indicated with asterisks.


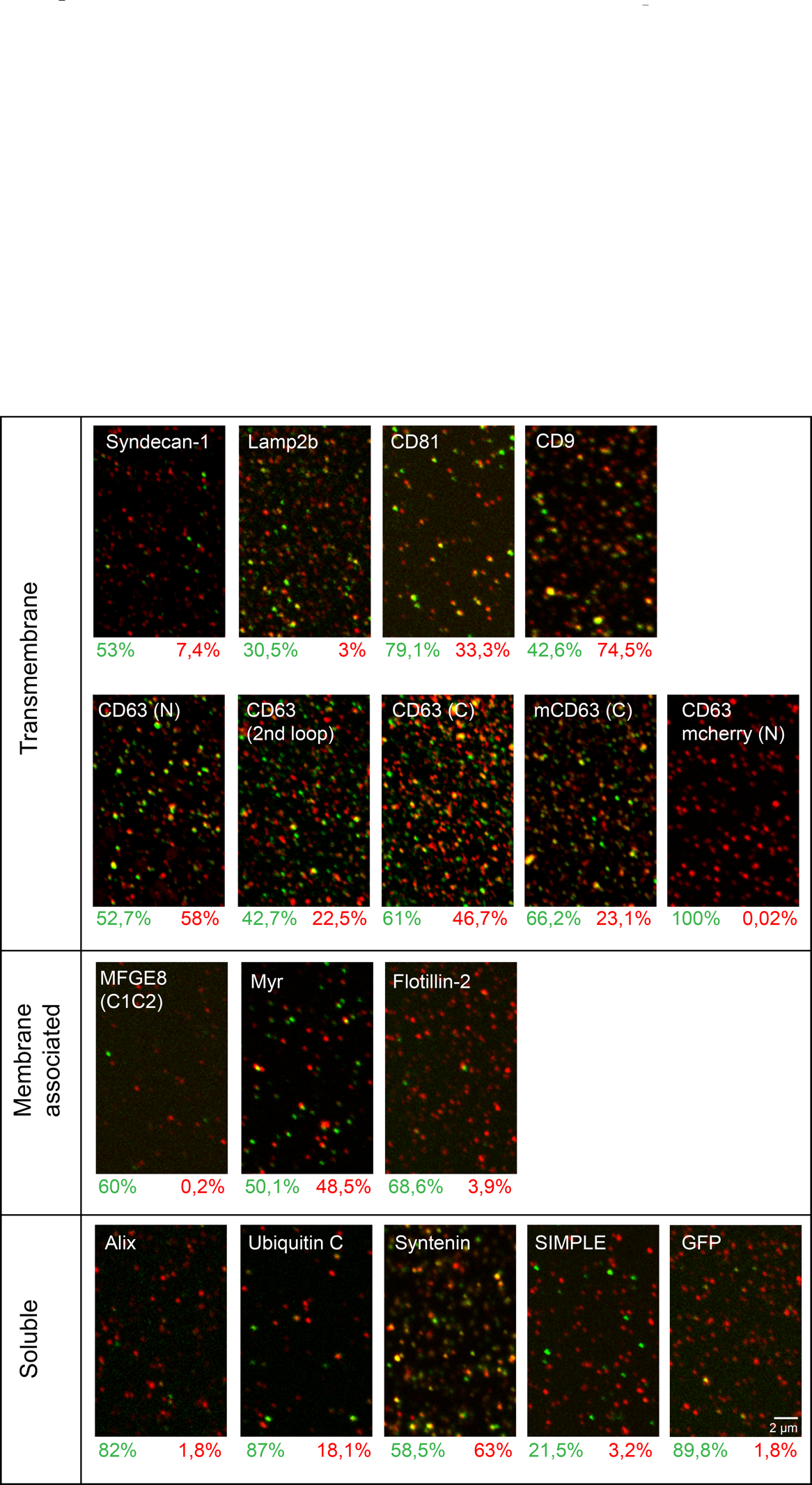


**Supplementary Figure 12.** Single vesicle imaging using confocal fluorescence microscopy after spotting EVs, double labelled with CD63-mCherry and each one of the GFP tagged EV sorting domains, onto coverslips. GFP and mCherry fluorescent vesicles were detected and the single images were overlapped in order to measure and quantify the colocalization rate. Colocalization percentage of GFP-tagged vesicles co-expressing CD63mCherry (green on the left) and CD63-mCherry vesicles co-expressing GFP proteins (red on the right) are depicted underneath each image. Scale bar 2 µm.


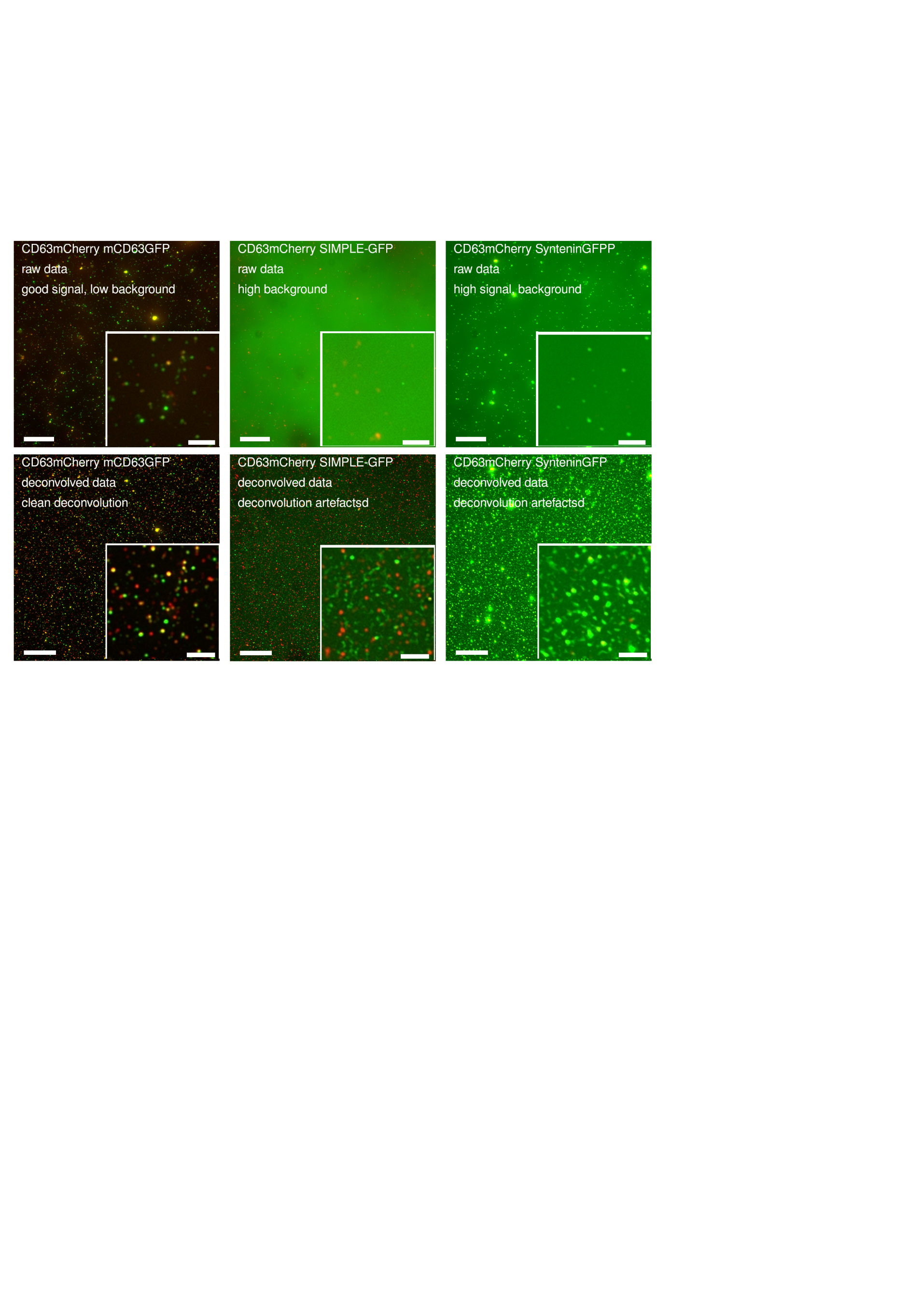


**Supplementary Figure 13.** Co-localization of different GFP-tagged EV proteins with CD63-mCherry EVs by single vesicle widefield imaging. Images are shown before (top row) and after deconvolution (bottom row) to highlight the green fluorescent background and show a deconvolution artefact. Due to this high fluorescent background in the CD63mCherry/SIMPLE-GFP and CD63mCherry/Syntenin-GFP samples, a comparable quantification to all the other samples was not possible.
