# Supplementary figures and images for: Systematic characterization of extracellular vesicle sorting domains and quantification at the single molecule – single vesicle level by fluorescence correlation spectroscopy and single particle imaging
Source: J Extracell Vesicles. 2019 Sep 18;8(1):1663043. doi: 10.1080/20013078.2019.1663043 (PMC6758720; doi:10.1080/20013078.2019.1663043)

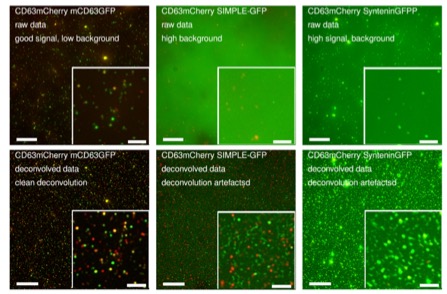

Supplement: Supplemental Material [file ZJEV_A_1663043_SM7244.zip › ZJEV_A_1663043_Supplementary/Supp.Fig. 13.jpg]
